# SOD1 activity threshold and TOR signalling modulate VAP(P58S) aggregation via reactive oxygen species-induced proteasomal degradation in a *Drosophila* model of amyotrophic lateral sclerosis

**DOI:** 10.1242/dmm.033803

**Published:** 2019-02-07

**Authors:** Kriti Chaplot, Lokesh Pimpale, Balaji Ramalingam, Senthilkumar Deivasigamani, Siddhesh S. Kamat, Girish S. Ratnaparkhi

**Affiliations:** 1 Department of Biology, Indian Institute of Science Education and Research, Pune 411008, India; 2 Oxford Nanoimaging Ltd, Oxford OX2 8TA, UK

**Keywords:** ALS, Autophagy, UPS, Aggregate, Rapamycin, MG132

## Abstract

Familial amyotrophic lateral sclerosis (ALS) is an incurable, late-onset motor neuron disease, linked strongly to various causative genetic loci. *ALS8* codes for a missense mutation, P56S, in VAMP-associated protein B (VAPB) that causes the protein to misfold and form cellular aggregates. Uncovering genes and mechanisms that affect aggregation dynamics would greatly help increase our understanding of the disease and lead to potential therapeutics. We developed a quantitative high-throughput *Drosophila* S2R+ cell-based kinetic assay coupled with fluorescent microscopy to score for genes involved in the modulation of aggregates of the fly orthologue, VAP(P58S), fused with GFP. A targeted RNA interference screen against 900 genes identified 150 hits that modify aggregation, including the ALS loci *Sod1* and *TDP43* (also known as *TBPH*), as well as genes belonging to the mTOR pathway. Further, a system to measure the extent of VAP(P58S) aggregation in the *Drosophila* larval brain was developed in order to validate the hits from the cell-based screen. In the larval brain, we find that reduction of SOD1 levels or decreased mTOR signalling reduces aggregation, presumably by increasing the levels of cellular reactive oxygen species (ROS). The mechanism of aggregate clearance is, primarily, proteasomal degradation, which appears to be triggered by an increase in ROS. We have thus uncovered an interesting interplay between SOD1, ROS and mTOR signalling that regulates the dynamics of VAP aggregation. Mechanistic processes underlying such cellular regulatory networks will lead to better understanding of the initiation and progression of ALS.

This article has an associated First Person interview with the first author of the paper.

## Abstract (precis)

**Summary:** ALS8/VAPB(P58S) aggregates appear to be cleared by an increase in cellular reactive oxygen species, which, in turn, triggers proteasomal degradation.

## INTRODUCTION

Amyotrophic lateral sclerosis (ALS) is a progressive, fatal neurodegenerative disease characterized by loss of motor neurons, resulting in muscular atrophy, gradual paralysis and, ultimately, death of the patient within 2-5 years post-diagnosis (Cleveland and Rothstein, 2001; Tarasiuk et al., 2012). Most often, the disease occurs sporadically (sporadic ALS). However, in ∼10% of the cases, the disease occurs due to inheritance of altered gene(s) (familial ALS). *SOD1* (also known as *ALS1*), coding for superoxide dismutase 1, was the first causative locus to be discovered (Deng et al., 1993; Rosen et al., 1993), with more than 170 SOD1 mutations attributed to the diseased state. Since then, about 50 potential genetic loci (Taylor et al., 2016) have been identified in ALS through genome-wide association, linkage and sequencing studies. Recent studies have emphasized the oligogenic basis for ALS (Deivasigamani et al., 2014; van Blitterswijk et al., 2012), suggesting that ALS loci may be a part of a gene regulatory network (GRN) that breaks down late in the life of a diseased individual. At the cellular level, several hallmarks of ALS include breakdown of cellular homeostasis (Cluskey and Ramsden, 2001), endoplasmic reticulum (ER) stress, unfolded protein response, aggregation, oxidative stress, mitochondrial dysfunction and autophagy. Although several studies have demonstrated the wide range of consequences of the genetic alterations on cellular function, no clear unifying mechanism has emerged that might explain the pathogenesis of the disease (Andersen and Al-Chalabi, 2011; Mulligan and Chakrabartty, 2013; Taylor et al., 2016; Turner et al., 2013; Walker and Atkin, 2011).

In 2004, Mayana Zatz's group (Nishimura et al., 2004) discovered a novel causative genetic locus, VAMP-associated protein B (VAPB), termed as ALS8, in a large Brazilian family whose members succumbed to ALS and/or spinal muscular atrophy. The point mutation of P56S was identified in the N-terminal, major sperm protein (MSP) domain of VAPB (Nishimura et al., 2004). VAPB is an integral membrane protein present in the ER membrane, ER-Golgi intermediate compartment, mitochondrial-associated membrane and the plasma membrane, implicated in important functions in the cell such as vesicular trafficking, ER structure maintenance, lipid biosynthesis, microtubule organization, mitochondrial mobility and calcium homeostasis (Lev et al., 2008; Murphy and Levine, 2016). Recent studies have highlighted its critical role in membrane contact sites (Alpy et al., 2013; Gomez-Suaga et al., 2017b; Metz et al., 2017; Yadav et al., 2018; Zhao et al., 2018). The *Drosophila* orthologue of VAPB is VAP33A/CG5014 (herein referred to as VAP) and has been used to develop models for ALS (Chai et al., 2008; Deivasigamani et al., 2014; Moustaqim-Barrette et al., 2014; Ratnaparkhi et al., 2008; Sanhueza et al., 2015). We have previously identified a *Drosophila* VAP gene regulatory network consisting of 406 genes, including a novel interaction with the mTOR pathway (Deivasigamani et al., 2014). The ALS8 mutation can also alter the physical interaction of VAP with other proteins, including FFAT motif-containing proteins (Loewen et al., 2003; Murphy and Levine, 2016), impairing cellular functions (De Vos et al., 2012; Huttlin et al., 2015; Moustaqim-Barrette et al., 2014). Ubiquitinated cellular aggregates (Papiani et al., 2012; Ratnaparkhi et al., 2008) are seen on VAP mutant expression and are capable of sequestering the wild-type VAP protein in a dominant-negative manner (Ratnaparkhi et al., 2008; Teuling et al., 2007). In *Drosophila*, neuronal overexpression of VAP(P58S), and subsequent formation of aggregates, in the background of endogenous VAP appears to lead to only mild neurodegenerative phenotypes, such as flight defects, compared with the more severe phenotypes associated with wild-type VAP neuronal overexpression (Ratnaparkhi et al., 2008; Tsuda et al., 2008). Previously, we have used the UAS-GAL4 system to study the interaction between VAP and mTOR signalling using the neuromuscular junction (NMJ) phenotype associated with neuronally overexpressed VAP(P58S) (Deivasigamani et al., 2014). The functional consequence of neuronal VAP(P58S) aggregation in this system is not fully understood, and its contribution to disease remains elusive.

In this study, we identify 150 genetic modifiers of VAP(P58S) aggregation by conducting a directed S2R+ cell-based RNA interference (RNAi) screen, targeting 900 unique genes belonging to different categories that are associated either with ALS or VAP function or proteostasis. We used the previously described [C155-Gal4;UAS-VAP(P58S)] system (Deivasigamani et al., 2014; Ratnaparkhi et al., 2008) to validate one such modifier, SOD1, *in vivo*, in the third-instar larval brain of *Drosophila*, by measuring changes in aggregation of VAP(P58S) in response to modulation of *Sod1* levels. Our data indicate that clearance of VAP(P58S) aggregates via the proteasomal machinery is enhanced by inducing reactive oxygen species (ROS) due to loss of SOD1 function. We also find a similar clearance of aggregates, attributed to proteasomal degradation, with mTOR downregulation, accompanied by elevated ROS. We find that wild-type VAP, but not mutant VAP, elevates ROS. Accumulated ROS result in inhibition of endogenous *VAP* transcription, a phenomenon that may directly affect familial as well as sporadic ALS pathogenesis.

## RESULTS

### A *Drosophila* S2R+ cell culture model to study VAP(P58S) aggregation

**Fig. 1.**
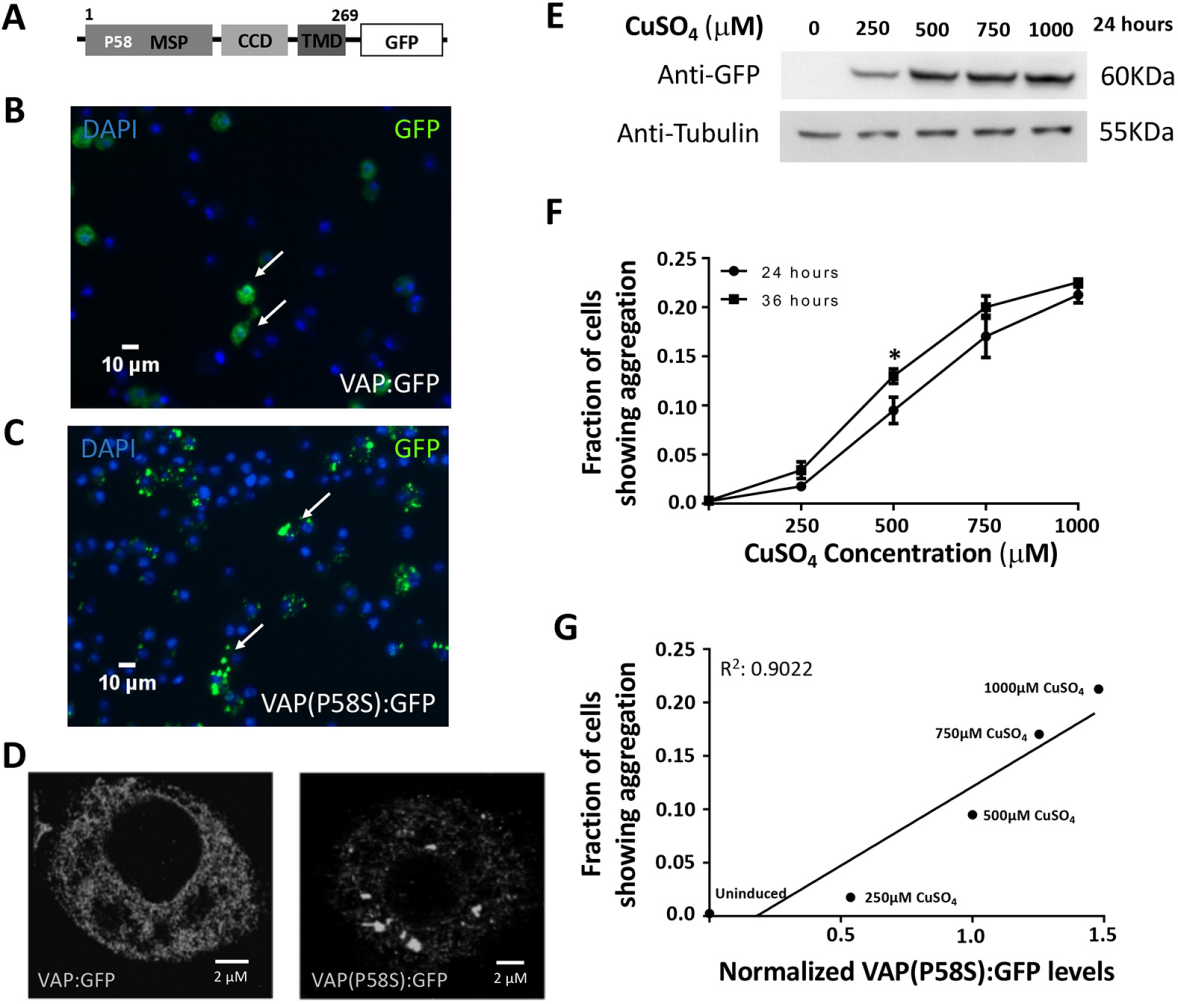
**A *Drosophila* cell culture model to study VAP(P58S) aggregation.** (A) VAP:GFP and VAP(P58S):GFP, when expressed in S2R+ cells, allow efficient visualization of VAP protein in the cell by epifluorescence. (B,C) In stable cell lines, expression of *VAP(P58S):GFP*, under an inducible metallothionein promoter results in aggregation (C), unlike wild-type VAP:GFP (B). GFP is visualized by epifluorescence and chromatin by DAPI, post-fixation. Arrows indicate cells expressing VAP:GFP (B) or VAP(P58S):GFP (C). (D) A super-resolution image, using Ground State Depletion microscopy, showing GFP inclusions formed in cells expressing VAP(P58S):GFP but not in VAP:GFP. (E) VAP(P58S):GFP protein levels in cells increase with increasing CuSO_4_ concentration at 24 h post-induction. (F) The increase in the fraction of S2R+ cells showing GFP-positive inclusions increases with increasing CuSO_4_ concentration. At 500 mM CuSO_4_, inclusions significantly increase between 24 h and 36 h. Student's *t*-test (*P<0.05). (G) A linear correlation between the fraction of cells showing aggregation, measured using microscopy, plotted against relative VAP(P58S):GFP protein levels, as quantified by western blotting, at 24 h post-induction. Error bars indicate s.d.

C-terminal and N-terminal fusions of VAP and VAP(P58S) with GFP were used to transfect cells and generate stable S2R+ lines, as described in the Materials and Methods (Fig. 1A; Fig. S1A). VAP:GFP showed a non-nuclear, reticular localization in the cell with <10% of the transfected (GFP-positive) cells showing high intensity puncta (Fig. 1B; Fig. S1A). In contrast, >80% of the GFP-positive VAP(P58S):GFP cells showed distinct high-intensity puncta with little or no background staining within the cell (Fig. 1C; Fig. S1A). Super-resolution imaging confirmed that VAP appeared to be reticular, while VAP(P58S) was found in inclusion bodies (Fig. 1D). In contrast, GFP, when expressed, showed a uniform cytoplasmic signal (Fig. S1B). Both N-terminal GFP fusions, GFP:VAP and GFP:VAP(P58S), showed puncta formation at levels comparable to VAP(P58S):GFP, and hence were not used further in the study (Fig. S1A). All further experiments (see below) were carried out with stable lines expressing VAP:GFP or VAP(P58S):GFP, which showed expected/relevant localization and levels of aggregation.

### An S2R+ cell-based reverse-genetics screen developed to identify modifiers of VAP(P58S) aggregation

In an attempt to identify genetic modifiers of VAP(P58S) aggregation kinetics, we conducted a focused S2R+ cell-based RNAi screen, targeting 900 unique genes belonging to nine different categories or families associated with ALS or VAP function. We generated stable S2R+ cell lines expressing VAP(P58S):GFP under a Cu^2+^-induced promoter. The inducible cell culture system allowed us to increase the VAP(P58S):GFP protein levels in the cell with increasing copper sulphate (CuSO_4_) concentrations (250, 500, 750 and 1000 μM) at 24 h post-induction (Fig. 1E). Using fluorescence microscopy, we found a linear relationship between the CuSO_4_ concentrations and the fraction of cells showing VAP(P58S):GFP aggregates that also increased with time (24 h and 36 h) post-induction (Fig. 1F). The concentration-dependent increase in relative levels of VAP(P58S):GFP correlated with an increase in the fraction of cells showing aggregates (Fig. 1G), indicating the propensity of the mutant protein to aggregate. Early time points (12-16 h) gave very few cells with aggregates, while non-linearity, high confluency and cell death became a concern at time points beyond 48 h and concentrations greater than 750 µM. The aggregation kinetics curve was used to define the extent of aggregation in the cell culture system and select optimum parameters to conduct the RNAi screen. Keeping a modest confluency and well-separated cells for ease of imaging, the screen was performed at a fixed concentration of 500 µM CuSO_4_ at 24 h and 36 h post-induction.

**Fig. 2.**
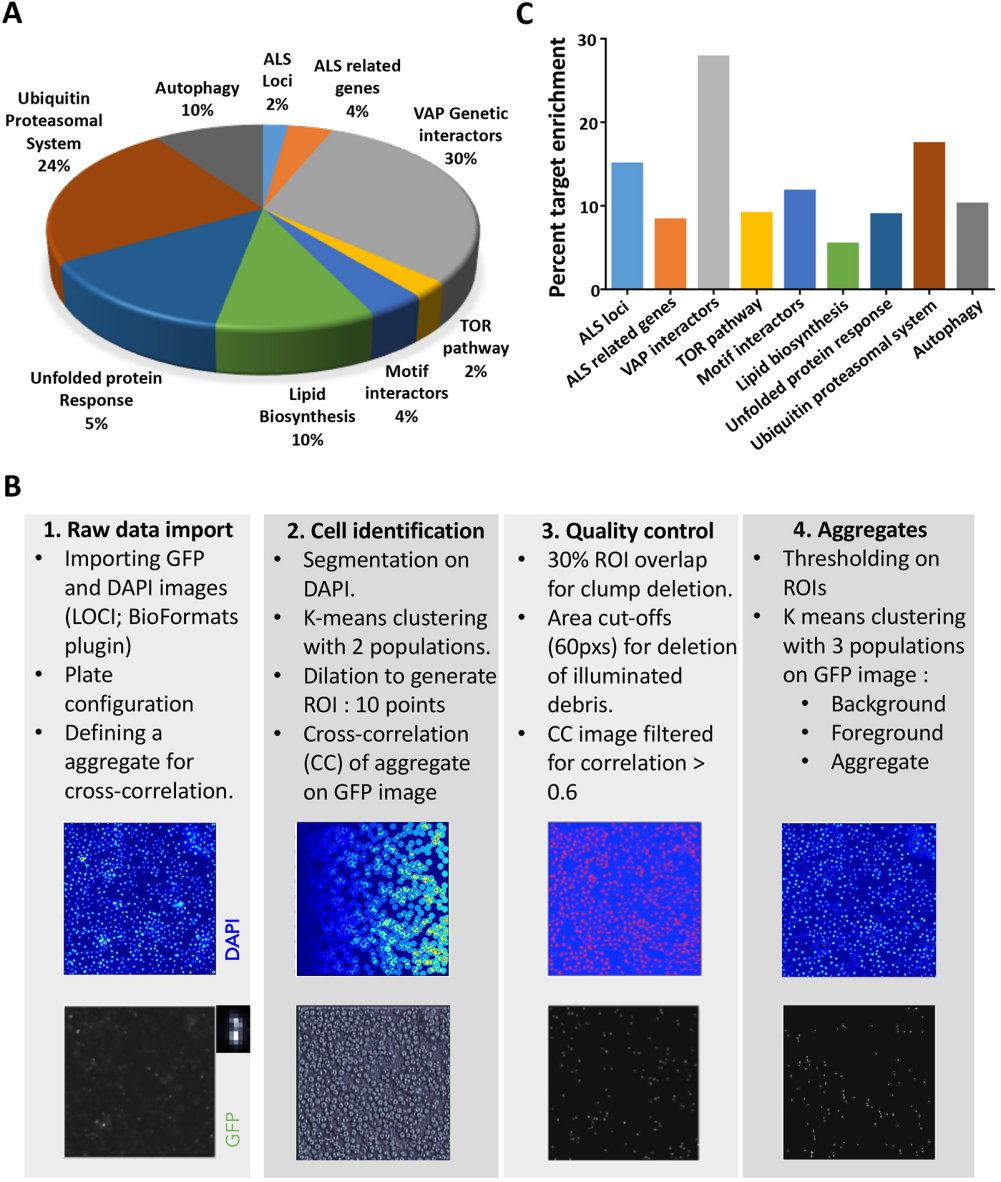
**A targeted dsRNA screen in S2R+ cells to discover modifiers of VAP(P58S):GFP aggregation.** (A) dsRNAs for 900 genes (Table S1A) were chosen for knockdown. GO representation indicates the categories of genes chosen and percentage (%) for each category. Genes were categorized as indicated (Table S1A,B). (B) Workflow of the steps executed for image analysis using an automated MATLAB script (Dey et al., 2014). Steps are detailed in the Materials and Methods. (C) The end result of the screen is a list of 150 genes identified, based on average cell intensity, which have been found to modify aggregation of VAP(P58S):GFP. Graph indicates the percentage of genes identified as targets within each gene category. Genes are listed in Table S1C*.*

We chose 900 genes (Table S1A), based on their availability in the Open Biosystems Library (see Materials and Methods), to screen for modifiers that could change aggregation levels of VAP(P58S):GFP. A Gene Ontology (GO) chart (Fig. 2A) represents the biological process associated with these 900 genes, as defined by FlyBase. The genes were selected and categorized (Table S1B) on the following basis. First, known *Drosophila* orthologues of ALS loci (20 genes) and ALS-related genes (36 genes) as tabulated in the online ALS database (ALSOD) were chosen. The next category included 273 genes from a VAP *Drosophila* GRN consisting of 406 genes (Deivasigamani et al., 2014). As *Mtor* was identified as a major interactor of *VAP* in our previous study (Deivasigamani et al., 2014), we chose 22 genes of the extended mTOR pathway. To explore the functional aspects of VAP(P58S), we also screened genes involved in lipid biosynthesis (92 genes) and FFAT motif interactors of VAP (34 genes). In order to identify a role of proteostasis in aggregation, we screened genes involved in the unfolded protein response (123 genes), ubiquitin proteasomal pathway (212 genes) and autophagy (88 genes).

The images collected at the end of the screen (detailed in the Materials and Methods) were analysed by an automated MATLAB analysis (see Materials and Methods; Fig. 2B). Based on average cell intensity, 150 targets (Table S1C) and, based on total cell intensity, 85 targets (Table S1D) that modulated VAP(P58S):GFP aggregation kinetics were identified; 57 genes were found to be overlapping for both parameters, increasing confidence in our analysis (Table S1E). The percentage of genes identified as modulators from each category are plotted in Fig. 2C and Fig. S1C, as percent target enrichment. ALS loci, notably *Sod1* and *TDP43* (also known as *TBPH*), were found as interesting modulators perturbing VAP(P58S):GFP aggregation. Targets belonging to the VAP genetic network, as defined by Deivasigamani et al. (2014), were also enriched. As identified earlier (Deivasigamani et al., 2014), components of the mTOR pathway also appeared to be key regulators of VAP(P58S):GFP aggregation. Less than 10% of genes screened belonging to families associated with lipid biosynthesis and motif interactors were identified as targets. Interestingly, genes related to the ubiquitin proteasomal system (UPS), such as ubiquitin ligases and proteasome components, were enriched, as were the autophagy-related genes, *Atg7* and *Atg3*. From the unfolded protein response category, along with chaperones such as the heat shock proteins *Hsp60C*, *Hsp23* and *Hsp83*, we also identified a few peptidyl prolyl isomerases as targets. Overall, in our primary targeted screen, we found various genetic interactors of wild-type VAP as modulators of VAP(P58S) aggregation as well. Importantly, the uncovering of two ALS loci, *Sod1* and *TDP43*, mTOR pathway genes such as *Rheb* and *S6k*, and genes enriched in the UPS as modulators of VAP(P58S) aggregation dynamics, led us to develop an *in vivo* model to validate these genes and to understand mechanisms underlying these interactions in *Drosophila*.

### A model system for measuring VAP(P58S) aggregation in the *Drosophila* larval brain

In order to validate targets from the screen *in vivo*, we used the *UAS-GAL4* system to specifically overexpress wild-type *VAP* or *VAP(P58S)* in the brain using a pan-neuronal driver, *C155* (*elav*) (Deivasigamani et al., 2014; Ratnaparkhi et al., 2008). Based on anti-VAP immunostaining, unlike wild-type VAP (Fig. S2A), mutant VAP(P58S) formed distinct cellular puncta and could be used as a model to study aggregation in the animal (Fig. S2B-D). These aggregates have been shown to be ubiquitinated and dominant negative when expressed in muscle (Ratnaparkhi et al., 2008). To develop a methodology for quantitation of aggregates in the brain (described in the Materials and Methods), we used temperature as a means to increase GAL4 activity, which would increase VAP(P58S) dosage and, possibly, aggregation. An increase in mean VAP(P58S) aggregation density was observed with increasing temperature, which was significant between 18°C and 25°C, but not significant between 25°C and 28°C (Fig. S2H). Neuronal knockdown of VAP, using RNAi, in *C155-GAL4/+; UAS-VAP(P58S)/+* flies, at each temperature (Fig. S2E-G), led to a significant decrease in the corresponding aggregation density of the ventral nerve cord (Fig. S2H). The above experiments suggested that, at 25°C, we could quantify changes in VAP(P58S) aggregation density in the brain of the larvae and, thereafter, we used this system to further validate modifiers of aggregation identified from the cell-based screen.

### *Drosophila* SOD1 is a modifier of VAP(P58S) aggregation

**Fig. 3.**
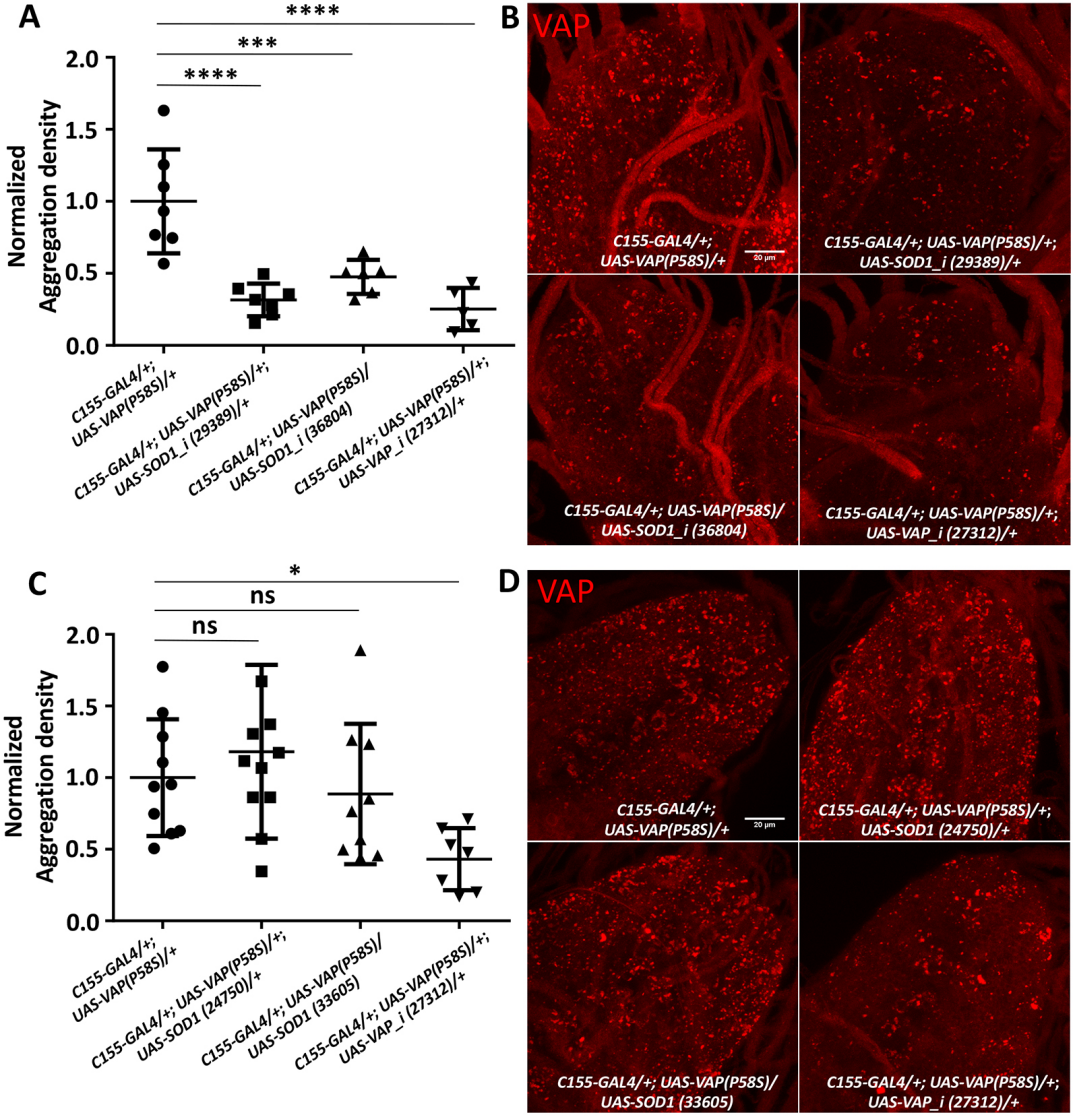
***Sod1* knockdown reduces VAP(P58S) aggregation in larval brains.** (A) *Sod1* knockdown in the nervous system decreases aggregation density in the ventral nerve cord. VAP knockdown also reduces aggregation due to reduction in VAP and VAP(P58S) protein expression. The ‘*_i* ’ appended to a gene name indicates an RNAi line. ANOVA (****P<0.0001). Numbers in brackets indicate BDSC stock numbers. (B) Representative images of the ventral nerve cord showing aggregation of VAP(P58S) with *Sod1* knockdown (29389 and 36804) and with *VAP* knockdown (27312). (C) *Sod1* overexpression does not affect aggregation density in the ventral nerve cord. ANOVA (*P*=0.0208). (D) Representative images of the ventral nerve cord showing aggregation of VAP(P58S) with *Sod1* overexpression (24750 and 33605) and with *VAP* knockdown (27312). All images were taken at the same magnification. Fisher's LSD multiple comparison (*P<0.05, ***P<0.001, ****P<0.0001; ns, not significant). Error bars indicate s.d.

*SOD1*, the first known ALS locus (Rosen et al., 1993), has been implicated in sporadic as well as familial cases and was our first choice for validation of the S2R+ based screen in *Drosophila*. We previously identified *Sod1* as a genetic interactor of *VAP* in a fly-based reverse genetics screen (Deivasigamani et al., 2014). Here, we individually knocked down *Sod1* using three independent RNAi lines in the *C155-GAL4/+; UAS-VAP(P58S)/+* background and observed a significant decrease in aggregation density in the ventral nerve cord (Fig. 3A,B; Fig. S3A,C,D). This threefold decrease in VAP aggregates was comparable to the reduction seen with VAP knockdown (Fig. 3B). Likewise, we overexpressed *Sod1* in the *C155-GAL4/+; UAS-VAP(P58S)/+* background. Here, however, we did not find a significant change in aggregation density (Fig. 3C,D; Fig. S3B,C,E). Taken together, these results suggest a need for a threshold level of *Sod1* to maintain VAP(P58S) inclusions.

### Oxidative stress reduces VAP(P58S) aggregation

**Fig. 4.**
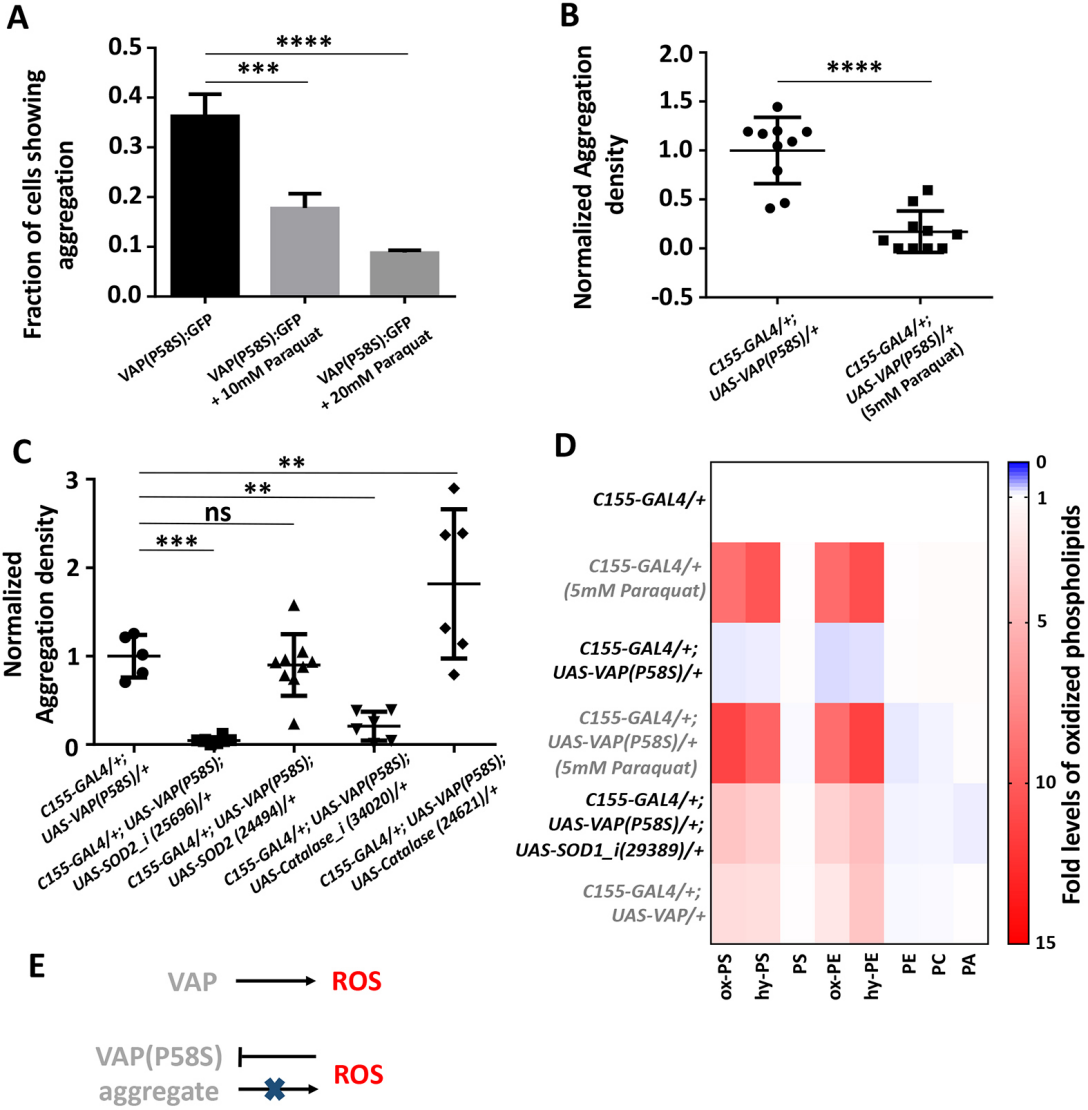
**Increase in ROS leads to decrease in VAP(P58S) aggregation levels.** (A) 4 h paraquat treatment prior to inducing VAP(P58S):GFP in stable S2R+ cell line reduces the fraction of cells showing aggregation observed 24 h post-induction. ANOVA (****P<0.0001), Fisher's LSD multiple comparison test (***P<0.001, ****P<0.0001). Representative images are shown in Fig. S4A. (B) Paraquat feeding decreases aggregation density in the ventral nerve cord of third-instar larval brains in *C155-GAL4/+; UAS-VAP(P58S)/+* flies. Student's *t*-test (****P<0.0001). Representative images are shown in Fig. S4B. (C) *Sod2* or *Catalase* knockdown reduces aggregation density. Overexpression of *Sod2* does not change aggregation density; however, overexpression of *Catalase* increases aggregation density. The ‘*_i* ’ appended to a gene name indicates an RNAi line. ANOVA (****P<0.0001), Fisher's LSD multiple comparison test (**P<0.01, ***P<0.001; ns, not significant). (D) Heat map depicting the change in levels of oxidized phospholipids normalized to *C155-GAL4/+*, quantified using MS in response to ROS generated in third-instar larval brains (*n*=4) for the listed genotypes. *Sod1* knockdown as well as VAP overexpression appears to increase cellular ROS levels. Statistical tests are described in Table S2. (E) Model depicting the effects of overexpression of wild-type and mutant VAP on ROS. Error bars indicate s.d.

Enzymatically, SOD1 metabolizes superoxide species to hydrogen peroxide, thereby preventing oxidative stress. A loss of function of SOD1 would, in principle, increase ROS. We tested whether a chemical mimic, paraquat, which increases cellular ROS (Castello et al., 2007; Cochemé et al., 2011; Drechsel and Patel, 2008), could phenocopy the effect of *Sod1* knockdown. We treated the VAP(P58S):GFP stable line with non-lethal concentrations of 10 mM and 20 mM paraquat for 4 h prior to CuSO_4_ induction and found that paraquat could significantly reduce the fraction of cells showing GFP-positive aggregates (Fig. 4A; Fig. S4A) in a dose-dependent manner. Similarly, larvae with the genotype *C155-GAL4/+; UAS-VAP(P58S)/+* hatched, fed and grown on a non-lethal concentration of 5 mM paraquat at 25°C showed a decrease in aggregation density in the third-instar larval brain, reminiscent of the *Sod1* knockdown phenotype (Fig. 4B; Fig. S4B). We also checked the effect of other ROS scavenging genes, such as *Sod2* and *Catalase*, on VAP(P58S) aggregation. Knockdown of both these genes resulted in a drastic reduction in aggregation density in the ventral nerve cord of *C155-GAL4/+; UAS-VAP(P58S)/+* larval brains (Fig. 4C). As seen with SOD1, overexpression of SOD2 did not change aggregation density; however, Catalase overexpression resulted in a fractional increase in aggregation density (Fig. 4C). These results strongly suggest ROS-dependent maintenance and/or stability of VAP(P58S) aggregates.

To confirm whether feeding of paraquat and loss of SOD1 function led to an increase in ROS levels in the larval brain, we measured the levels of oxidized phospholipids, using quantitative mass spectrometry (MS)-based lipidomics (Kamat et al., 2015; Kory et al., 2017; Pathak et al., 2018; Tyurina et al., 2000). On feeding *C155-GAL4/+* larvae with 5 mM paraquat, we enriched and detected nine oxidized polyunsaturated fatty acids (PUFAs), belonging to the phosphatidylserine (PS) and phosphatidylethanolamine (PE) (Fig. 4D; Table S2) families of phospholipids, which were significantly elevated in larval brains, compared with the unfed control. PUFA-containing oxidatively damaged phospholipids showed a mass addition of +16 (denoted as ‘ox-’, likely an epoxide across the double bond) or +18 (denoted as ‘hy-’, likely the addition of water across the double bond) to the parent phospholipid, as a consequence of addition of different ROS. Of note, the parent or precursor phospholipids did not change in concentration, and the concentrations of the oxidized phospholipids were less than 1% of the parent or precursor phospholipids. We found a similar elevation in concentrations of oxidized phospholipids in *C155-GAL4/+; UAS-VAP(P58S)/+; UAS-SOD1_i/+*, but not in *C155-GAL4/+; UAS-VAP(P58S)/+*, which was equivalent to the *C155-GAL4/+* control (Fig. 4D; Table S2). This elevation in oxidized phospholipids was found to be inversely correlated with the corresponding fold change in aggregation density (Fig. S4C). Interestingly, we found that overexpression of *VAP* had a curious effect of increasing the oxidation of lipids, indicating that wild-type VAP has a cryptic, yet important, role in regulating ROS levels. Taken together, these results indicate that ROS initiate processes that aid clearance of VAP(P58S) aggregates and are, in turn, regulated by VAP wild-type levels in the cell (Fig. 4E).

### ROS activate proteasomal machinery

**Fig. 5.**
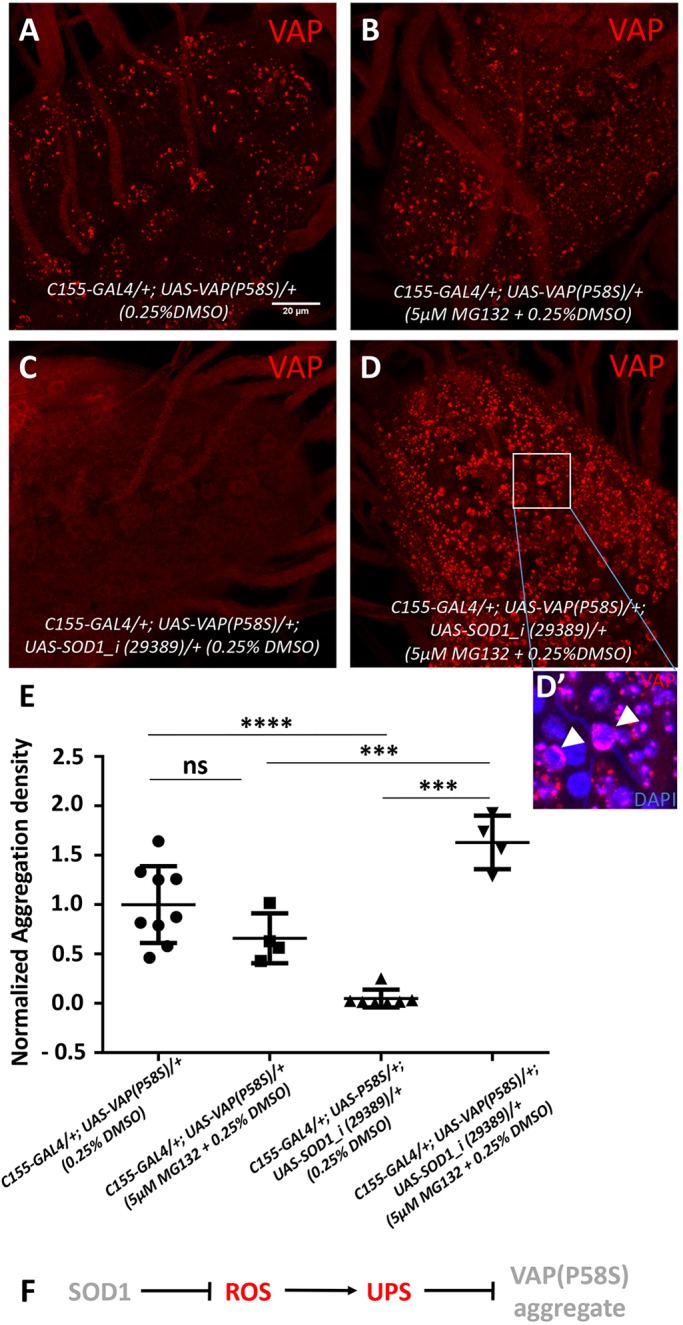
**ROS activates proteasomal machinery.** (A,B) MG132 feeding of *C155-GAL4/+; UAS-VAP(P58S)/+*, to inhibit proteasomal machinery, does not accumulate VAP aggregates. (C-D′) MG132 feeding of *C155-GAL4/+; UAS-VAP(P58S)/+; UAS-SOD1_i (29,389)/+*, leads to a dramatic accumulation of VAP aggregates. The aggregates, in the presence of ROS and MG132, seem to be localized around the nuclear membrane (arrowheads) as depicted in the inset (D′). (E) Plot showing a significant decrease in aggregation density in the ventral nerve cord in *C155-GAL4/+; UAS-VAP(P58S); UAS-SOD1_i (29,389)/+* compared with *C155-GAL4/+; UAS-VAP(P58S)/+* control. This decrease is rescued by feeding 5 µM MG132 and is significantly higher than that in the *C155-GAL4/+; UAS-VAP(P58S)/+* control, both unfed and fed with MG132. All images were taken at the same magnification. ANOVA (****P<0.0001), Fisher's LSD multiple comparison test (***P<0.001, ****P<0.0001; ns, not significant). (F) Model depicting the role of SOD1-regulated ROS in activating proteasomal degradation of VAP(P58S) protein/aggregates. Error bars indicate s.d.

We further investigated protein degradative mechanisms that may be activated in response to ROS, leading to the clearance of VAP(P58S) aggregates. In order to test whether the proteasomal machinery was responsible for reduction in aggregation, we hatched, fed and grew larvae on food containing a proteasomal inhibitor, 5 µM MG132. Larval brains were dissected at the wandering third-instar stage and analysed for aggregation density. As expected, unfed *C155-GAL4/+; UAS-VAP(P58S)/+; UAS-SOD1_i/+* showed reduced aggregation density (Fig. 5C), compared with unfed control (Fig. 5A,E). Upon MG132 feeding, *C155-GAL4/+; UAS-VAP(P58S)/+; UAS-SOD1_i/+* showed a complete recovery/retention of VAP(P58S) aggregation (Fig. 5D,E). Fed *C155-GAL4/+; UAS-VAP(P58S)/+; UAS-SOD1_i/+* also showed an enhanced aggregation density compared with fed *C155-GAL4/+; UAS-VAP(P58S)/+* (Fig. 5B,E)*.* Aggregates in the presence of ROS (with *Sod1* knockdown) and proteasomal inhibition (with MG132) appeared to be predominantly smaller, scattered and mislocalized around the nuclear membrane/ER compared with the respective controls (Fig. 5D′). The localization of the aggregates suggests that they may be residing in a juxtanuclear quality control compartment (JUNQ)-like compartment (Ogrodnik et al., 2014). These results indicate that the proteasomal machinery is facilitated in the presence of ROS for active degradation of VAP(P58S) aggregates (Fig. 5F). However, fed *C155-GAL4/+; UAS-VAP(P58S)/+* larvae (Fig. 5A) did not show accumulation of aggregation, compared with unfed control (Fig. 5B,E), indicating that other mechanisms could be at play to maintain the aggregation density.

### mTOR downregulation, but not autophagy, lowers VAP(P58S) aggregation

**Fig. 6.**
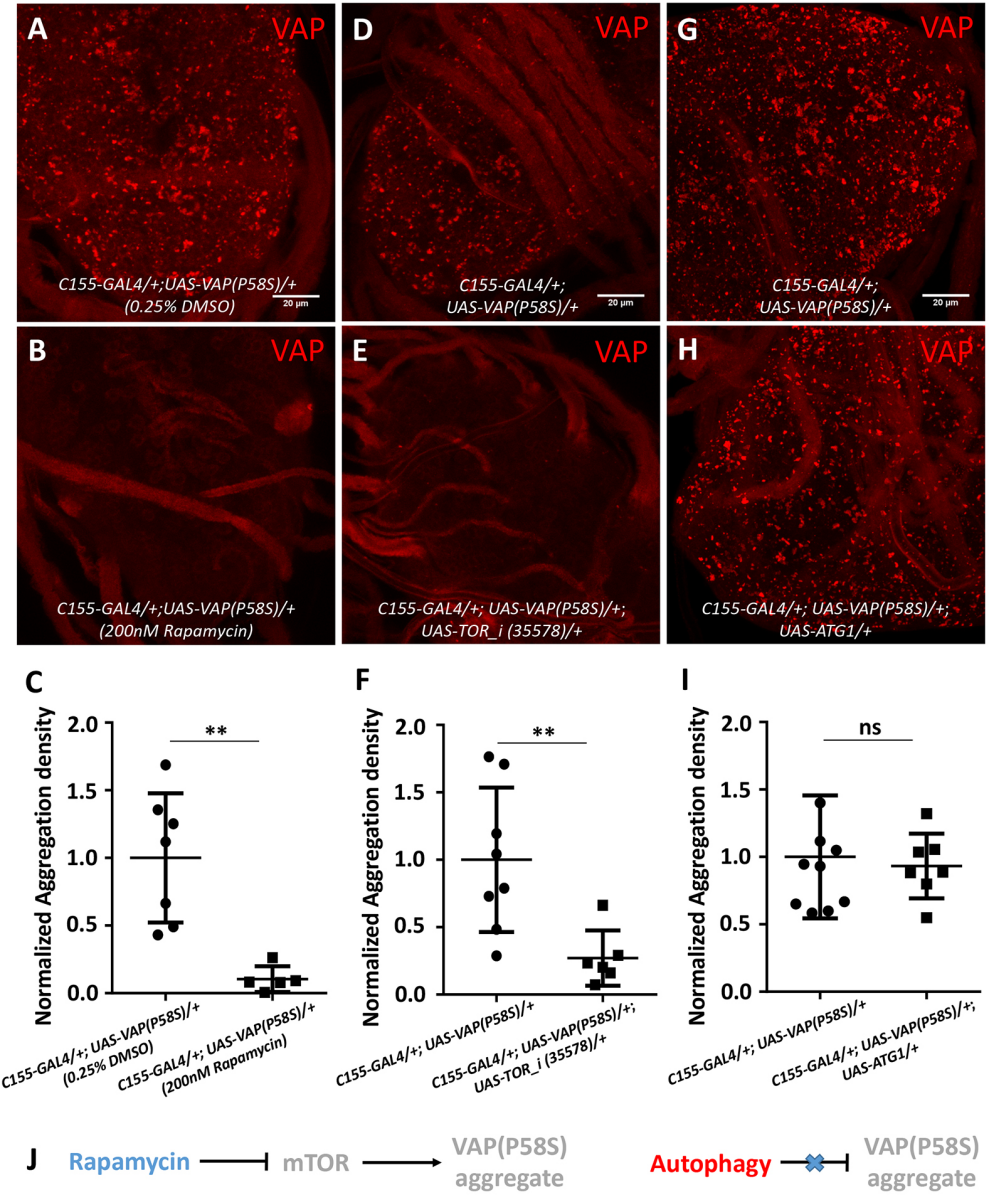
**mTOR downregulation, but not autophagy, reduces VAP(P58S) aggregation.** (A-C) Rapamycin feeding decreases aggregation density in the ventral nerve cord of third-instar larval brains in *C155-GAL4/+; UAS-VAP(P58S)/+* flies. (D-F) Neuronal *Tor* knockdown decreases aggregation density in the ventral nerve cord. The ‘*_i* ’ appended to a gene name indicates an RNAi line. (G-I) Neuronal overexpression of Atg1 did not affect the aggregation density in the ventral nerve cord. All images were taken at the same magnification. Student's *t*-test (**P<0.01; ns, not significant). (J) Model depicting mTOR-regulated clearance of aggregates, independent of autophagy. Error bars indicate s.d.

We examined whether aggregates could be cleared via autophagy in the third-instar larval brain. mTOR downregulation is known to activate autophagy (Noda and Ohsumi, 1998), and this could be achieved chemically, by feeding rapamycin (Heitman et al., 1991), and genetically, by *Tor* knockdown. Upon feeding *C155-GAL4/+; UAS-VAP(P58S)/+* larvae with 200 nM rapamycin as described before (Deivasigamani et al., 2014), we observed a drastic clearance of aggregates in the ventral nerve cord compared with unfed controls (Fig. 6A-C). When *Tor* transcripts were reduced using RNAi in *C155-GAL4/+; UAS-VAP(P58S)/+*, a similar decrease in aggregation density was found (Fig. 6D-F). To verify the effect of mTOR downregulation on aggregates, we induced autophagy by overexpressing Atg1 in *C155-GAL4/+; UAS-VAP(P58S)/+* larval brains as described before (Deivasigamani et al., 2014; Shen and Ganetzky, 2009). Validation of the UAS-Atg1 line is described in the Materials and Methods. With overexpression of Atg1, however, we did not observe a change in aggregation density (Fig. 6G-I; Fig. S4D,E), suggesting that mTOR signalling might perturb downstream effectors other than Atg1, which may affect VAP(P58S) aggregation dynamics (Fig. 6J). The data also raise the possibility of an autophagy-independent pathway.

### mTOR inhibition promotes proteasomal clearance of VAP(P58S) aggregation via ROS

**Fig. 7.**
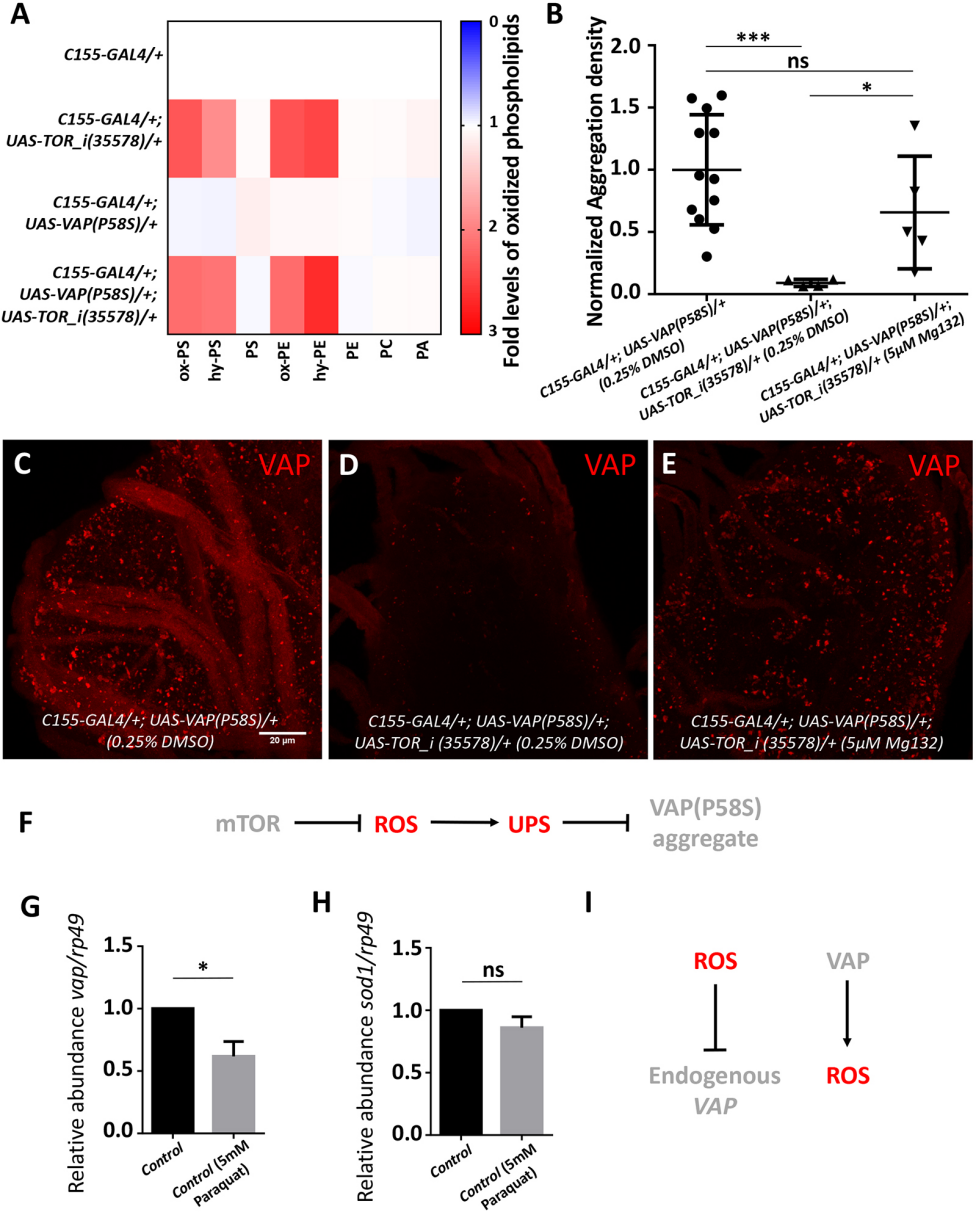
**mTOR inhibition induces ROS and promotes proteasomal degradation of VAP(P58S) protein/aggregates.** (A) Heat map depicting change in levels of oxidized phospholipids with *Tor* knockdown normalized to *C155-GAL4/+*, quantified using MS in response to ROS generated in third-instar larval brains (*n*=3-4) for the listed genotypes. Statistical tests are described in Table S2. (B) Plot showing a significant decrease in aggregation density in the ventral nerve cord in *C155-GAL4/+; UAS-VAP(P58S); UAS-TOR_i (35,578)/+* compared with *C155-GAL4/+; UAS-VAP(P58S)/+* control. This decrease is partially rescued by feeding 5 µM MG132. ANOVA (*P*=0.0042), Fisher's LSD multiple comparison test (*P<0.05, ***P<0.001; ns, not significant). (C-E) Representative images of third-instar larval brains showing the partial recovery of aggregates upon 5 µM MG132 feeding in *C155-GAL4/+; UAS-VAP(P58S)/+; UAS-TOR_i (35,578)/+* larvae. All images were taken at the same magnification. (F) Model depicting the role of mTOR-regulated ROS in activating proteasomal degradation of VAP(P58S) protein/aggregates. (G) Relative mRNA levels of *VAP* in the *C155-GAL4* control larval brain are lowered upon feeding animals 5 mM paraquat, suggesting that high levels of ROS may negatively regulate *VAP* transcripts. Student's *t*-test (*P<0.05). (H) Relative mRNA levels of *Sod1* in the *C155-GAL4* control larval brain do not change upon feeding 5 mM paraquat (ns, not significant). (I) Model depicting the differential relationship of ROS with VAP. Error bars indicate s.d.

We first decided to check whether clearance of aggregates with mTOR inhibition correlated with an increase in ROS, as in the case of *Sod1* knockdown. We found that levels of several species of oxidized phospholipids were indeed higher with *Tor* knockdown, with or without neuronal overexpression of VAP(P58S), in third-instar larval brains, with levels similar to those observed upon *Sod1* knockdown (Fig. 7A). mTOR pathway downregulation has recently been shown to activate not only autophagy but also ubiquitin proteasomal machinery (Zhao et al., 2015) via the Mpk1/ERK5 (also known as MAPK7) pathway in yeast and humans (Rousseau and Bertolotti, 2016). We tested whether ROS upregulation with *Tor* knockdown could be inducing proteasomal clearance of VAP(P58S) aggregation by feeding *C155-GAL4/+; UAS-VAP(P58S)/+; UAS-TOR_i/+* with 5 µM MG132 (Fig. 7B-E). Although there was a significant decrease in aggregation density with *Tor* knockdown (Fig. 7D), we found only a slight recovery of aggregation in MG132-fed animals (Fig. 7E) compared with unfed *C155-GAL4/+; UAS-VAP(P58S)/+* control larvae (Fig. 7C). This recovery appeared to be far less dramatic than that seen in the case of *Sod1* knockdown. Taken together, these results indicate that, in the context of ROS, proteasomal degradation could be the major pathway responsible for clearance of VAP(P58S) aggregation (Fig. 7F), although other downstream effectors of mTOR signalling, including autophagy, cannot be conclusively ruled out as additional mechanisms.

We also explored the possible relationship between *VAP* and ROS at a transcriptional level. Larvae of the control, *C155-GAL4/+* genotype were hatched and fed on 5 mM paraquat, and the brains were dissected at the wandering third-instar larval stage. The levels of endogenous *VAP* and *Sod1* mRNA, in response to ROS, were measured using quantitative PCR in control larval brains. We found that endogenous *VAP* mRNA levels were lowered in the presence of high levels of ROS (Fig. 7G), whereas *Sod1* mRNA levels remained unchanged (Fig. 7H). This result may indicate the presence of a negative-feedback loop wherein VAP overexpression leads to accumulation of ROS (Fig. 4C), which, in turn, downregulates endogenous *VAP* transcription (Fig. 7I). This phenomenon merits detailed investigation in future studies.

## DISCUSSION

### A targeted RNAi screen uncovers SOD1, TDP43 and TOR signalling elements as targets to understand dynamics of VAP(P58S) aggregation

*Drosophila* S2R+ cell-based whole-genome RNAi screens serve as powerful tools due to the relative ease with which transcript knockdown can be achieved (Echeverri and Perrimon, 2006). Similar systems have been used for identifying modifiers of aggregation of Huntingtin protein (Zhang et al., 2010). Our screen was aimed at enriching genes that are known players in ALS, VAP interactors and proteostasis. First and foremost, we found ALS loci *Sod1* and *TDP43* as modifiers of VAP(P58S) aggregation, which we had previously identified as VAP genetic interactors (Deivasigamani et al., 2014). In this study, we have explored the interaction between *Sod1* and *VAP*, while *TDP43* also serves as an exciting candidate for further investigation. TDP43 has been shown to perturb membrane-associated mitochondrial (Turner et al., 2008) sites that are maintained by VAPB-PTPIP51 interactions in mammalian cell culture (Stoica et al., 2014). Additionally, TDP43 proteinopathy has been identified in motor neurons of mice models of VAP(P58S) aggregation (Tudor et al., 2010). TDP43-driven neurodegeneration has also been shown to be modulated by oxidative stress-related MAP kinase pathways in a *Drosophila* screen (Zhan et al., 2015) and associated with the Nrf2 (also known as Nfe2l2)-dependent antioxidant pathway (Moujalled et al., 2017). In addition to *Sod1*, we have also identified other ROS-related genes – such as *peroxiredoxin V*, *NADH dehydrogenase*, *cytochrome c oxidase* – coding for proteins that localize to the mitochondria, perturbation of which will lead to oxidative stress, potentially affecting the aggregation kinetics of VAP(P58S).

Second, we enriched a subset of targets involved in protein degradation, the UPS and autophagy, an *in vivo* validation of which would shed light on the how these aggregates are compartmentalized and managed in the neurons. Third, this screen highlighted specific chaperones that could be involved in the misfolding and formation of VAP(P58S) aggregates, providing insight into the initiation of the disease condition. Most importantly, through our previous study (Deivasigamani et al., 2014), and our cell-based screen followed by subsequent experimentation, we have established mTOR signalling as a strong modulator of VAP(P58S) aggregation. mTOR signalling responds and integrates signals from nutrients, growth factors, energy and stress, and regulates cellular proteostasis, thus contributing to age-related neurodegenerative diseases (Perluigi et al., 2015), making it an attractive target for further investigation in ALS pathogenesis. Indeed, rapamycin, a mTORC1 inhibitor, is now being used for phase-II clinical trials for ALS (Mandrioli et al., 2018). Lastly, through our screen, targeting processes involved in neurodegeneration, we have identified interactions that point towards a role for VAP as a contributor to a common GRN, in agreement with several examples in the literature (Deivasigamani et al., 2014; Paillusson et al., 2017; Prause et al., 2013; Stoica et al., 2014, 2016; Tudor et al., 2010; van Blitterswijk et al., 2012). When we compared our list of targets with the results from another fly-based screen for VAP(P58S)-induced eye degeneration (Sanhueza et al., 2015), we only found one overlap, *Atg7*, a gene coding for a E1-like ubiquitin-activating enzyme with a role in autophagy (Mizushima and Komatsu, 2011). This lack of significant overlap could possibly be because of differences in sets of genes screened, cell types and phenotypes visualized.

### A ROS-dependant physiological mechanism that triggers proteasomal clearance of VAP(P58S) aggregation

**Fig. 8.**
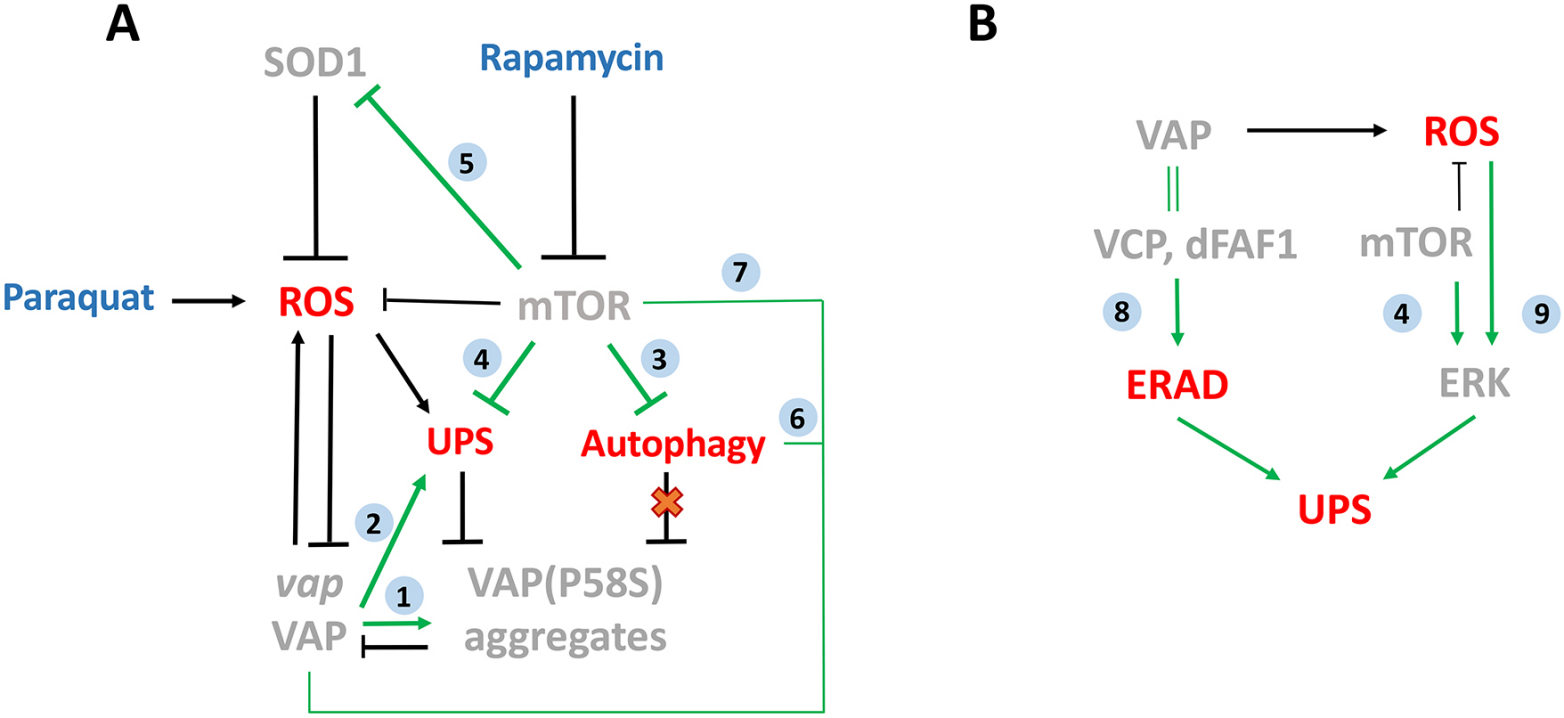
**An integrated model for ROS mediated clearance of VAP(P58S) aggregates via UPS.** (A) Model depicting novel relationships of SOD1- and mTOR-induced ROS with VAP and VAP(P58S) aggregates. Clearance of VAP(P58S) protein/aggregates appears to be primarily via the UPS, triggered by ROS, which are, in turn, regulated by cellular pathways such as the mTOR pathway, SOD1 and VAP activity. Autophagy does not appear to be a major contributor to aggregate clearance, under the conditions of our experiment. (B) A hypothetical model proposing the possible link between VAP, ROS and UPS. VAP could regulate the UPS via the ERAD pathway due to its interaction with VCP via dFAF1/Caspar. ROS could be the connecting link between the mTOR pathway and ERK pathway, which together regulate the components of the proteasomal machinery. The link between VAP and ROS that we have demonstrated could modulate proteasomal activity in the cell. Gray italic text, gene; gray upper-case non-italic text, proteins; red text, cellular mechanisms; blue text, drugs; black arrows, experimental evidence/this study; green arrows, relationship described in the literature. Numbers in blue circles indicate research papers: (1) Ratnaparkhi et al., 2008; (2) Kanekura et al., 2006; Kuijpers et al., 2013; (3) Noda and Ohsumi, 1998; Perluigi et al., 2015; (4) Zhao et al., 2015; Rousseau and Bertolotti, 2016; (5) Sun et al., 2012; Tsang et al., 2018; (6) Gomez-Suaga et al., 2017a,b; Zhao et al., 2018; Wu et al., 2018; (7) Deivaisigamani et al., 2014; (8) Baron et al., 2014; Papiani et al., 2012; (9) Cavanaugh et al., 2006; Su et al., 2014.

In our study, we have used a dosage-dependent pan-neuronal GAL4 expression of VAP(P58S) in order to study changes in aggregation in the third-instar larval brain. We found two targets, SOD1 and mTOR (Deivasigamani et al., 2014), the downregulation of which led to a decrease in VAP(P58S) aggregation accompanied by oxidative stress. We identified a role of ROS in upregulating the proteasomal machinery, thereby facilitating the degradation of misfolded VAP(P58S) protein/aggregates (integrated model, Fig. 8A). However, in the absence of ROS, we did not find any change in aggregation density upon pharmacological proteasomal inhibition. This is consistent with the cell culture studies that point towards the downregulation of the UPS due to VAP(P58S) aggregation, signifying a dominant-negative effect on wild-type VAP function (Genevini et al., 2014; Gkogkas et al., 2008; Kanekura et al., 2006; Papiani et al., 2012). Overexpression of VAP(P58S), or loss of VAP, in *Drosophila* has been shown to enhance ER stress in the adult brain and might be a result of suspended proteasomal degradation (Moustaqim-Barrette et al., 2014; Tsuda et al., 2008). In mice, VAP(P56S) aggregates have been shown to represent an ER quality control compartment that develops as a result of a debilitated ER-associated degradation (ERAD) pathway (Kuijpers et al., 2013). Indeed, VAP has been shown to interact with the unfolded protein response sensor AFT6 in mice and the ERAD complex, thereby regulating proteostasis and lipid homeostasis in HeLa cell lines (Gkogkas et al., 2008; Ernst et al., 2016). Studies in mammalian cell lines suggest that VAP(P56S) is ubiquitinated, aggregates on the ER membrane and is cleared by the AAA+ valosin-containing protein (VCP)/p97, which interacts with Fas-associated factor 1 (FAF1) and may use the FFAT motif in FAF1 as an adapter to interact with VAP (Baron et al., 2014; Papiani et al., 2012). In *Drosophila*, VAP has been shown to be essential for ER homeostasis by maintaining lipid transport, whereas the mutant VAP flies show accumulation of ubiquitinated and membrane proteins in neuronal cells (Moustaqim-Barrette et al., 2014). Hence, although ER stress is built up with VAP(P58S) aggregation, it does not lead to subsequent oxidative stress, as shown in our results. This suggests that ROS enhances the proteasomal degradation of VAP(P58S) through an ER stress-independent mechanism. Although neuronal VAP(P58S) aggregates appeared to be non-toxic to flies, our study highlights the effects of ROS on the dynamics of VAP(P58S), from misfolded protein to aggregate formation and subsequent clearance.

### TOR signalling regulates VAP(P58S) dynamics by UPS-dependent and Atg1-independent mechanisms

We previously identified the mTOR pathway as a strong regulator of both VAP and VAP(P58S) phenotypes at the NMJ (Deivasigamani et al., 2014). Here, we have shown that inhibition of the mTOR pathway also reduces VAP(P58S) aggregation levels in third-instar larval brains in the presence of ROS. mTOR pathway downregulation is known to activate autophagy (Noda and Ohsumi, 1998), a process that has been shown to reduce mutant huntingtin fragments (Ravikumar et al., 2004) and amyloid-β levels (Spilman et al., 2010) in mice models. The role of VAP in autophagy is unclear. With *VAP* (also known as *Vapb*) knockdown in mammalian cell culture, autophagy is upregulated due to the loss of calcium homeostasis that arises with the disruption of ER-mitochondrial contact sites (Gomez-Suaga et al., 2017a,b). This upregulation appears to be dependent on beclin-1, which has a role in autophagosome formation (Wu et al., 2018). However, VAP is also suggested to have a role in autophagosomal biogenesis through direct interaction with the ULK1/FIP200 (also known as RB1CC1) complex (Zhao et al., 2018). Previously, we have observed that neuronal overexpression of VAP or Atg1 reduces bouton size at the NMJ, an effect that is exacerbated in combination (Deivasigamani et al., 2014). On the other hand, Atg1 overexpression rescues the large bouton size associated with VAP(P58S) overexpression in the third-instar larval brains (Deivasigamani et al., 2014). In this study, however, we do not observe any clearance of VAP(P58S) aggregates with overexpression of Atg1 alone (Fig. 8A).

*Mtor* and *Sod1* have been shown to be genetic interactors in *Drosophila*, with mTOR inhibition enhancing the lifespan defect incurred with *Sod1* knockdown (Sun et al., 2012). Recently, mTOR has been directly shown to regulate SOD1 activity by its phosphorylation based on nutrient availability in yeast and mammalian cells (Tsang et al., 2018). Although this phosphorylation site does not appear to be conserved in *Drosophila*, this study demonstrates the role of mTOR pathway in regulating ROS via SOD1. mTOR inhibition, specifically, mTORC1, has also been shown to activate proteasomal degradation independent of its other targets, such as 4EBP, S6K and Ulk (Cavanaugh et al., 2006; Zhao et al., 2015). An evolutionarily conserved regulation of components of proteasomal assembly by mTORC1 via Mpk1/ERK5 has been reported in yeast and mammalian cell culture (Rousseau and Bertolotti, 2016). ERK5 signalling has been implicated in neuroprotective roles in response to mild levels of oxidative stress (Cavanaugh et al., 2006; Su et al., 2014). These studies suggest that ROS regulation by mTOR inhibition via SOD1 and ERK5 serves as a plausible mechanism for the proteasomal degradation of VAP(P58S) protein/aggregation and, by extension, the rescue of the VAP(P58S) NMJ phenotype (Deivasigamani et al., 2014) (Fig. 8B).

### Increase in ROS by VAP, but not VAP(P58S), expression

SOD1-associated elevation in ROS levels and oxidative stress is suggested as a plausible factor of motor neuron death in ALS (Barber et al., 2006; Saccon et al., 2013). Teuling et al. (2007) have shown that VAPB protein levels decrease in an age-dependent manner in a mouse model of SOD1-G93A, providing the first evidence of a link between *Sod1* and *VAP/Als8.* We now find that overexpressed VAP, unlike VAP(P58S), promotes the accumulation of ROS in the system. This is consistent with a study that shows lowered ROS in a *vpr-1* (VAP orthologue) mutant of *Caenorhabditis* *elegans* in response to increased mitochondrial connectivity and altered function (Han et al., 2012). VAP neuronal overexpression in *Drosophila* has also been shown to increase bouton number (Pennetta et al., 2002) similar to the SOD1 mutant phenotype at the NMJ (Milton et al., 2011), and is correlated with increased ROS in both scenarios. VAP may be important in regulating pathways that respond to changes in ROS levels, such as mTOR and ERK pathways that can regulate the UPS (Rousseau and Bertolotti, 2016). VAP also modulates ERAD (and the UPS), via its interaction with VCP and FAF1 (Baron et al., 2014; Papiani et al., 2012). We hypothesize that the interaction between VAP and ROS could lead to crosstalk between these pathways, regulating global proteostasis (hypothetical model, Fig. 8B).

### ROS may regulate VAP levels by regulating VAP transcription

In our study, we have found that, in the presence of ROS, *VAP* transcription is downregulated in wild-type flies. We had previously shown that *Sod1* knockdown rescues the VAP macrochaetae phenotype (Deivasigamani et al., 2014), which may be a consequence of excessive ROS accumulation, and subsequent downregulation of VAP levels and function. Two independent studies (Kim et al., 2016; Qiu et al., 2013) that overexpressed VAPB in *Sod1* (SOD1-G93A) mice, as an attempt at rescuing ALS defects, found contradictory observations, owing mainly to differences in expression levels of the protein. *VAPB* mRNA levels are known to be lowered in the spinal cords of patients with sporadic ALS (Anagnostou et al., 2010), as well as in induced pluripotent stem cell-derived motor neurons from ALS8 patients (Mitne-Neto et al., 2007). Based on our results, and taking into consideration earlier observations (Anagnostou et al., 2010; Deivasigamani et al., 2014; Teuling et al., 2007), we propose that a possible ALS disease scenario could include increased ROS, resulting in downregulation of VAP at the transcript level (integrated model, Fig. 8A). It remains to be tested whether ROS-activated pathways, such as MAP kinase pathways or the mTOR pathway, could directly control VAP expression. This VAP/ROS regulation that we have uncovered could have significant implications in ALS pathogenesis for both sporadic and familial ALS.

In summary, we find that the dynamics of VAP(P58S) neural aggregates in *Drosophila*, a species intimately linked to disease in the human context, is sensitive to levels of ROS. Change in the physiological levels of ROS appear to dictate the equilibrium between the aggregated and non-aggregated forms. The cellular levels of ROS are themselves dictated by well-characterized regulatory mechanisms that include ROS generators and scavengers. As shown in this study, TOR signalling and VAP/VAP(P58S) expression levels would contribute to the extent of aggregation, and may act as regulatory feedback loops to regulate physiological ROS levels. SOD1, VAP/ALS8, TOR and ROS appear to be part of a physiological regulatory circuit that maintains levels of VAP(P58S) aggregates.

## MATERIALS AND METHODS

### Generation of constructs and dsRNA

The complementary DNA (cDNA) sequences of VAP and VAP(P58S) mutant were cloned into *pRM-GFP* plasmid (Bhaskar et al., 2000) to generate both N- and C-terminal GFP fusions, using the *Eco*R1 restriction site. The pRM-GFP vector has GFP cloned into pRM-HA3 vector at the *Bam*HI site. We used 500 μM CuSO4 to drive expression in S2R+ cells after transient transfections. Double-strand RNA (dsRNA) for the secondary screen was generated using a MEGAscript^®^ T7 Kit (AM1333) by Thermo Fisher Scientific. The template for dsRNA was generated using cDNA as a template, prepared from flies. Primers for the template were ordered from Sigma-Aldrich.

### Handling of Schneider cells

*Drosophila* S2R+ cells, a kind gift from Dr Satyajit Mayor [National Centre for Biological Sciences (NCBS), Bangalore, India] were maintained in Schneider cell medium (21720-024, Gibco) with 10% heat-inactivated fetal bovine serum (FBS; 10270, Gibco). Batches of cells were frozen in 10% dimethyl sulfoxide (DMSO; D2650, Sigma-Aldrich) and stored in liquid nitrogen following the DRSC protocol (http://www.flyrnai.org/DRSC-PRC.html). In general, after reviving, cells were discarded after 25-30 passages. Cells were maintained at 23°C and split every 4 days at a ratio of 1:5.

### Cell culture and generation of S2R+ stable lines

Stable S2R+ cell lines were generated by co-transfecting with pRM-HA3 constructs of VAP:GFP, VAP(P58S):GFP or GFP along with pCo-Hygro in 20:1 ratio, using Effectene (Qiagen) and/or Mirus TransIT 2020 (MIR 5400), and selected under 250 µg/ml hygromycin (Sigma-Aldrich) for 10-15 passages. Stable as well as transiently transfected cell lines were induced to express the gene of interest under a metallothionein promoter using increasing concentrations (250, 500, 750 and 1000 μM) of CuSO_4_ and analysed at 12, 24, 36 and 48 h post-induction. Transient transfection assays were performed using Mirus TransIT-2020 (MIR 5400) transfection reagent. The protocol for the dsRNA knockdown assay was modified from Rogers and Rogers (2008). Fixation, 4′,6-diamidino-2-phenylindole (DAPI) staining and imaging were performed using an EVOS FL Auto Cell Imaging system. Super-resolution images of fixed VAP:GFP and VAP(P58S):GFP cells were acquired using a Leica SR GSD 3D system.

### Western blotting

Cells were centrifuged at 604 ***g*** for 5 min in an Eppendorf 5414R centrifuge. The pellet was resuspended in 20 μl supernatant and boiled with 1× SDS dye at 95°C. Samples were centrifuged again at 12,045 ***g*** for 10 min. Cell extracts were separated by 12% SDS-PAGE and transferred onto 0.45 μm polyvinylidene fluoride membrane (Millipore). Membranes were blocked for 1 h in 5% skimmed milk in 1× TBS containing 0.1% Tween 20 at room temperature, and probed with 1:10,000 diluted mouse anti-Tubulin (T6074, Sigma-Aldrich) and 1:5000 diluted mouse anti-GFP (Roche Life Science) overnight at 4°C (12 h). Anti-rabbit and anti-mouse secondary antibodies conjugated to horseradish peroxide (Pierce) were used at a dilution of 1:10,000 for 1 h at room temperature. Blots were developed with Immobilon Chemiluminescent Substrate (LuminataClassico Western HRP substrate from Millipore) using a LAS4000 Fuji imaging system.

### S2R+ cell culture imaging and analysis

Cell culture images were taken using 20× air objective DAPI (405 nm) and GFP (488 nm) channels to image nuclei and GFP-tagged protein/aggregates in each field, respectively, using an EVOS FL Auto Cell Imaging System. DAPI and GFP channel images were processed using ImageJ 1.48V. Macro scripts were recorded to quantify the total number of cells and number of cells showing aggregates. Total numbers of cells were quantified by converting the DAPI channel image to 8-bit, subtracting the measured mean intensity to remove background, converting greyscale to Binary, using watershed function for segmentation, and analysing particles of size 10-500 and circularity 1. Number of cells showing aggregates were quantified by converting the GFP channel image to 8-bit. Rolling ball background subtraction with 0.3 radius was used to integrate aggregates belonging to the same cell, based on proximity, as one object; the image was converted to Binary, and objects of size 10-500 were counted using ‘analyze particles’ tool.

### GO analysis

The list of genes and GO information was obtained based on FlyBase (http://flybase.org) (Marygold et al., 2013) entries. Genes were categorized manually in the broad categories of ALS genes, VAP interactome (Deivasigamani et al., 2014) and proteostasis. Lists of ALS loci and ALS-related genes were obtained from ALSOD (http://alsod.iop.kcl.ac.uk) (Wroe et al., 2008). The *Drosophila melanogaster* homologues of these ALS genes were identified using Ensembl biomart tool (http://asia.ensembl.org/biomart/martview) and FlyBase batch download tool. Human orthologues of the target genes listed in Table S1C-E were identified using FlyBase batch download tool.

### High-throughput screen and image acquisition

The screen was performed at the screening facility at the Centre for Cellular and Molecular Platforms (C-CAMP), NCBS (http://ccamp.res.in/HTS-HCI). dsRNA for the high-throughput screen was generated and plated into sixteen 384-well plates by Chromous Biotech (Bangalore, India) in preparation for the experiment. The library used as a template for generating dsRNAs was procured from Open Biosystems (RDM1189 and RDM4220). Cells (50 μl; 3×10^6^/ml) were plated in each well for the 384-well flat-bottom plates obtained from Corning. Each target dsRNA knockdown experiment was performed in triplicate, randomly arranged in the 384-well plate. The cells were treated with 10 μg/ml dsRNA for 48 h, followed by induction with 500 μM CuSO_4_. The cells were fixed and imaged at 24 h and 36 h post-induction with CuSO_4_. Fixation was performed with 4% paraformaldehyde in 1× PBS, after which cells were washed twice with 1× PBS, treated with 0.05 µg/ml DAPI and washed twice with 1× PBS. Each plate contained seven negative controls occupying 42 wells, and 114 unique genes were screened in each plate. A few genes were kept as overlap between multiple plates to check for their consistency and reproducibility. Imaging for the high-throughput screen was performed by an Array Scan VTI HCS system (Thermo Fisher Scientific). Dual-channel images from ten fields in each well were captured using a 20× air objective and an EMCCD camera. The fluorescein isothiocyanate (FITC; 488 nm) channel was used for imaging VAP(P58S):GFP aggregates, and the DAPI (405 nm) channel was used for imaging cell nuclei. Ten fields were imaged in each well and ∼400 cells were imaged per field. In well triplicates, ∼12,000 cells were imaged for each dsRNA knockdown.

### High-throughput data analysis

Images from the FITC and DAPI channels in each site were read using the Bio-Formats MATLAB toolbox (Linkert et al., 2010) and were processed using custom MATLAB scripts (Dey et al., 2014). The segmentation was performed using the DAPI images, and the extraction of pixel intensities was done on the FITC channel. Illumination correction was performed as a pre-processing step on the DAPI images, and individual nuclei were segmented after a contrast stretching routine was applied. The identified objects were further filtered for outliers, based on a size-based cutoff, and the individual eight connected components were labelled as separate nuclei. Under 20× magnification, we estimated the cellular radius to be ∼10 pixels, corresponding to 5 μm. Thus, labelled cellular objects (ROIs) were obtained by dilating the centroids of each nuclei by 10 pixels. Around 400 ROIs were obtained from each field, consistent with manually counted cells in these images. The resultant ROIs were further filtered for clumps and out-of-focus objects. The GFP intensities were obtained for these ROIs following a local background correction of the FITC images (with a disk size of 3 pixels). Average and total intensities were calculated from the pixel data obtained from every cell/ROI from these FITC images. A Kolmogorov–Smirnov-like statistic was used to assign *Z*-scores to each gene on plate as reported by Dey et al. (2014). A statistically significant threshold was obtained for the triplicate data using Monte Carlo simulations. Genes were classified as hits if they occurred two or more times above a given *Z*-score threshold. The false-positive rate for both parameters at both time points was zero. The false-negative rate for average intensity for the 24-h time point was 0.2523 and for the 36-h time point was 0.361. The false-negative rate for total intensity for the 24-h time point was 0.3838 and for the 36-h time point was 0.3164.

### Fly husbandry and brain aggregation assay

*D. melanogaster* lines were maintained on standard corn meal agar medium. *UAS-GAL4* system (Brand and Perrimon, 1993) was used for overexpression of transgenes. *UAS-VAP* wild type, *UAS-VAP(P58S)* and *C155-GAL4* lines used for fly experiments have been described earlier (Deivasigamani et al., 2014; Ratnaparkhi et al., 2008). Canton S flies were used as wild-type control. *UAS-VAP_i* (27312), *UAS-SOD1_i* (34616, 29389, 36804) and *UAS-TOR_i* (35578) (where the suffix ‘­*_i*’ indicates an RNAi line), and *UAS-SOD1* (24750, 33605), were obtained from Bloomington *Drosophila* Stock Centre (BDSC). Clone for UAS-FLAG-HA-tagged SOD1 in pUASt vector was obtained – for expression in *Drosophila* – from *Drosophila* Genome Research Centre (DGRC) and injected in the NCBS C-CAMP transgenic facility. Two independent *UAS-Atg1* lines were used for our experiments. One line (Mohseni et al., 2009; Scott et al., 2007) was procured from BDSC (51654), while the other was kindly provided by Dr Chen (Academia Sinica, Taipei, Taiwan). Both lines were validated in the wing and thorax using *ptc-GAL4* as described (Chen et al., 2008). Briefly, expression of the two Atg1 lines in the *ptc* domain results in missing anterior cross veins and loss of thoracic bristles. Additionally, expression of both lines using *actin-GAL4* also caused early lethality. Atg1 overexpression in the larval brain using BDSC 51654 has been shown to increase LysoTracker staining in the larval brain hemisphere, indicating activation of autophagy (Shen and Ganetzky, 2009). The readout of autophagy in our experiments is thus indirect and not based on specific cellular markers. For all genetic crosses, experiments were set at 18°C, 25°C or 28°C, as indicated. Brains were dissected from third-instar larvae and processed for immunostaining assay. For fixation, 4% paraformaldehyde containing 0.1% Triton X-100 was used, followed by washes with 1× PBS. Blocking treatment and washes were performed with 0.3% Triton X-100 with 2% bovine serum albumin. Brains were stained with 1:500 diluted anti-VAP antibody (Yadav et al., 2018) and 1:1000 anti-rabbit secondary (Invitrogen) was used. *Z*-stacks of five to ten brains for each sample were imaged under a 63× oil objective of a Ziess LSM 710 confocal microscope. The number of aggregates were quantified per μm^3^ of the ventral nerve cord, defined as ‘aggregation density’, using the Huygen professional software. The high-intensity puncta were considered as aggregates. An arbitrary threshold was set for controls as well as for test samples that achieved removing low-intensity background signal emitted by the tissue, along with separation of high-intensity puncta that were adjacent to one another. An object filter was used to remove objects of size greater than 1000 pixels, and garbage size smaller than 10 pixels was excluded. Three 3D region of interests of fixed size were drawn along the tip of the ventral nerve cord and the number of aggregates were counted from each of these ROIs and averaged for each animal. The volume (in μm^3^) of ROI depicting the thickness of the brain tissue was measured as the range of the *z*-stack of the image. The aggregation density obtained for each brain was normalized to the mean of the control group, *C155-GAL4; UAS-VAP(P58S)* (+0.25% DMSO, in the case of DMSO-soluble drug experiments) and plotted as ‘normalized aggregation density’ in each graph. Student’s *t*-test and one-way ANOVA with Fisher's least significance difference (LSD) multiple comparison test were used to measure statistical significance using GraphPad Prism 7.

### Drug treatment

Cells were exposed to 10 mM and 20 mM paraquat dichloride hydrate (500 mM, 36541, Sigma-Aldrich) for 24 h prior to protein induction with 500 µM CuSO_4_. Fixation, DAPI staining and imaging were performed using an EVOS FL Auto Cell Imaging System. For flies, 10-12 virgins were placed with CS males for each genotype, and animals were allowed to mate for 24 h and transferred to standard cornmeal fly medium containing paraquat (5 mM), MG132 (5 µM), rapamycin (200 nM) or DMSO (0.25%).

### Lipid extraction and targeted LC-MS lipidomics

All MS quantitation phospholipid standards were purchased from Avanti Polar Lipids (Alabaster, AL, USA). The brain samples were washed with PBS (three times), and transferred into a glass vial using 1 ml PBS. Then, 3 ml of 2:1 (vol/vol) CHCl_3_: MeOH with the internal standard mix (1 nmol 17:1 free fatty acids, 100 pmol each of 17:0-20:4 PS, 17:0-20:4 phosphatidylcholine, 17:0-20:4 PE and 17:0-20:4 phosphatidylalanine) was added, and the mixture was vigorously vortexed. The two phases were separated by centrifugation at 2800 ***g*** for 5 min. The organic phase (bottom) was removed, 50 μl formic acid was added to acidify the aqueous homogenate (to enhance extraction of phospholipids) and CHCl_3_ was added to make up 4 ml volume. The mixture was vortexed and separated using centrifugation as described above. Both the organic extracts were pooled and dried under a stream of N_2_. The lipidome was re-solubilized in 200 μl of 2:1 (vol/vol) CHCl_3_: MeOH, and 20 μl was used for the targeted liquid chromatography (LC)-MS analysis. All the phospholipid species analysed in this study were quantified using the multiple reaction monitoring (MRM) high-resolution scanning method on a Sciex X500R QTOF LC-MS with an Exion-LC series quaternary pump. All data were acquired and analysed using SciexOS software as described before (Pathak et al., 2018). LC separation was achieved using a Gemini 5U C-18 column (Phenomenex, 5 μm, 50×4.6 mm) coupled to a Gemini guard column (Phenomenex, 4×3 mm, Phenomenex security cartridge). The LC solvents were as follows: for positive mode: buffer A, 95:5 (vol/vol) H_2_O: MeOH+0.1% formic acid+10 mM ammonium formate; and buffer B, 60:35:5 (vol/vol) iPrOH: MeOH: H_2_O+0.1% formic acid+10 mM ammonium formate; for negative mode: buffer A, 95:5 (vol/vol) H_2_O: MeOH+0.1% ammonium hydroxide; and buffer B, 60:35:5 (vol/vol) iPrOH: MeOH: H_2_O+0.1% ammonium hydroxide. All the MS-based lipid estimations was performed using an electrospray ion source, using the following MS parameters: ion source=turbo spray, collision gas=medium, curtain gas=20 l/min, ion spray voltage=4500 V, temperature=400°C. A typical LC run consisted of 55 min, with the following solvent run sequence post-injection: 0.3 ml/min of 0% buffer B for 5 min, 0.5 ml/min of 0% buffer B for 5 min, 0.5 ml/min linear gradient of buffer B from 0 to 100% over 25 min, 0.5 ml/min of 100% buffer B for 10 min, and re-equilibration with 0.5 ml/min of 0% buffer B for 10 min. A detailed list of all the species targeted in this MRM study, describing the precursor parent ion mass and adduct, and the product ion targeted, can be found in Table S2. All the endogenous lipid species were quantified by measuring the area under the curve in comparison to the respective internal standard, and then normalizing to the number of larval brains. All oxidized phospholipids detected were normalized to the corresponding non-oxidized phospholipid internal standard. All data are represented as mean±s.e.m. of at least four biological replicates per genotype.

### mRNA isolation, cDNA preparation and quantitative reverse transcription PCR

Approximately 1 µg mRNA was isolated from 12-18 third-instar larval brains using a Direct-zol™ RNA MicroPrep Kit (R2062) from Zymo Research. The cDNA reaction was carried out using a High Capacity cDNA Reverse Transcriptase Kit (4368814) by Applied Biosystems. The quantitative PCR reaction was carried out using KAPA SYBR FAST (KK4602) by Sigma-Aldrich and Replex Mastercycler by Eppendorf. The experiment was carried out in three biological replicates with technical triplicates.

### Regulatory oversight

All experimental protocols were considered and approved by the Indian Institutes of Science Education and Research (IISER) Institutional Biosafety Committee (IBSC). The IBSC is overseen by the Review Committee on Genetic Manipulation, Department of Biotechnology, Government of India.

## Supplementary Material

Supplementary information
