# Supplementary material for: SOD1 activity threshold and TOR signalling modulate VAP(P58S) aggregation via reactive oxygen species-induced proteasomal degradation in a Drosophila model of amyotrophic lateral sclerosis
Source: Dis Model Mech. 2019 Feb 7;12(2):dmm033803. doi: 10.1242/dmm.033803 (PMC6398501; doi:10.1242/dmm.033803)
Supplement: Supplementary information [file dmm-12-033803-s1.pdf]

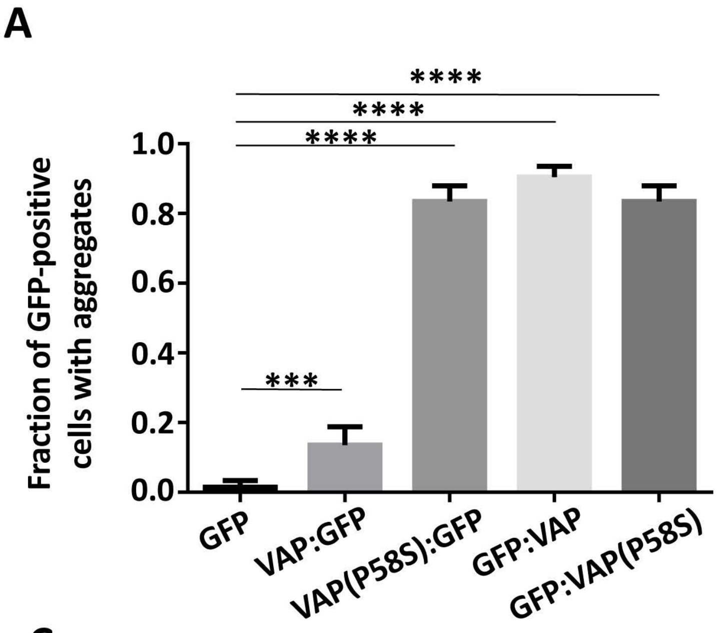

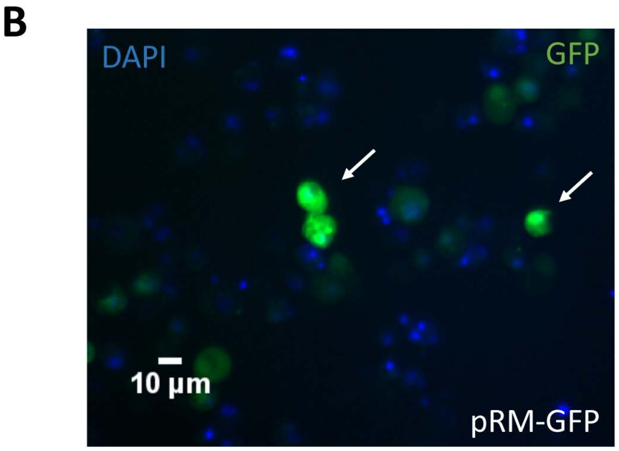

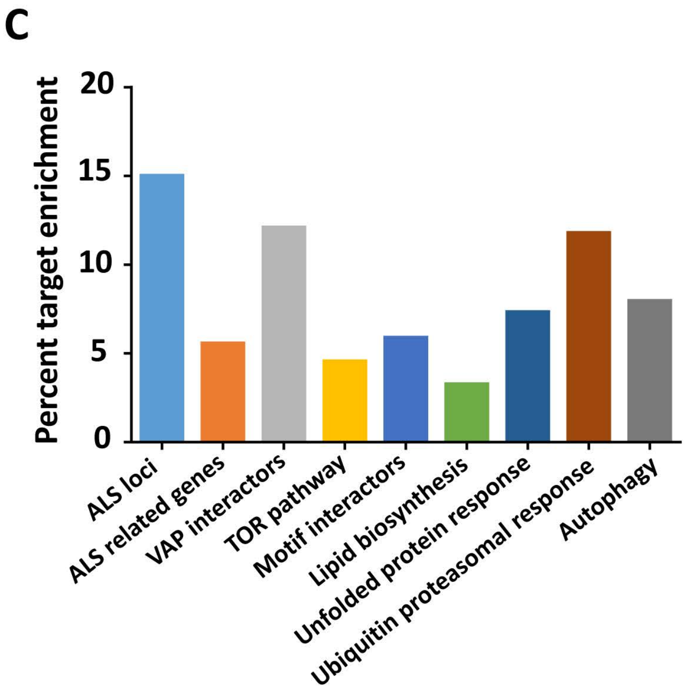

FIGURE S1:

A: Fraction of GFP-positive cells showing aggregates plotted for S2R+ cells transiently transfected with C-terminal or N-terminal tagged GFP constructs of VAP or VAP(P58S) as also only GFP construct at 24 hours post 500µM $CuSO_4$ induction. Unlike C-terminal tagged VAP, N-terminal tagged VAP forms, mutant and wild type, both aggregate. as GFP, when expressed alone does not aggregate or form puncta. ANOVA (P-value: ****<0.0001) Fisher's LSD multiple comparison test (P-values, ***<0.001, ****<0.0001).

B: Homogenous cytoplasmic expression of GFP in S2R+ cells.

C: A list of 85 genes identified based on total cell intensity as a parameter. Based on the analysis of the S2R+ screen, these genes modify aggregation of VAP(P58S):GFP. Graph displays the percent fold enrichment of targets within each gene category. Genes are listed in *Suppl. Table 1D.*

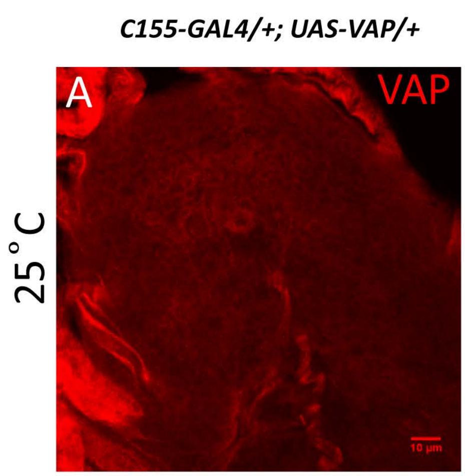

FIGURE S2: A system for measuring VAP(P58S) aggregation in the larval brain.

A: Overexpression of VAP in the ventral nerve cord of the third instar larval brain, driven by pan-neuronal *C155-GAL4*, immunostained with rabbit anti-CCD (VAP) antibody, shows membrane localization.

B-D: Overexpression of VAP(P58S) is visualized as inclusions in the third instar larval brains. Temperature dependent increase in aggregation density is seen in the ventral nerve cord in *C155-GAL4/+; UAS-VAP(P58S)/+* larvae.

E-G: Knockdown of *VAP* in *C155-GAL4/+; UAS-VAP(P58S)/+* larvae leads to a corresponding decrease in aggregation density at each temperature.

H: Plot showing significant increase in VAP(P58S) aggregation density with increase in temperature, and a significant decrease in aggregation density in the ventral nerve cord in *C155-GAL4/+; UAS-VAP(P58S); UAS-VAP_i (27312)/+* as compared to *C155-GAL4/+; UAS-VAP(P58S)/+* control in a temperature dependent manner.

All images were taken at the same magnification. ANOVA (P-value: ****<0.0001)Fisher's LSD multiple comparison test (P values, *<0.05, **<0.01, ***<0.001).

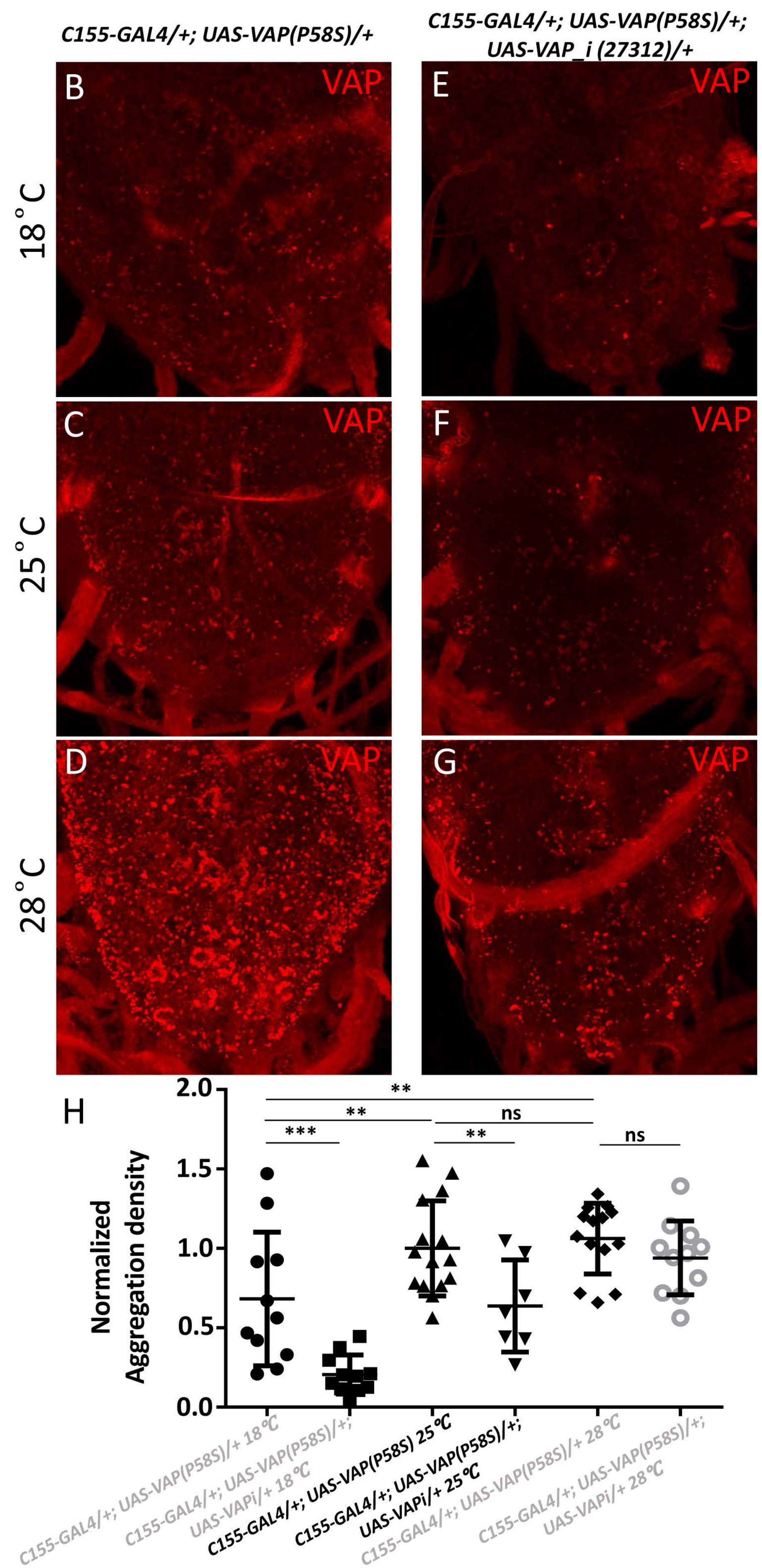

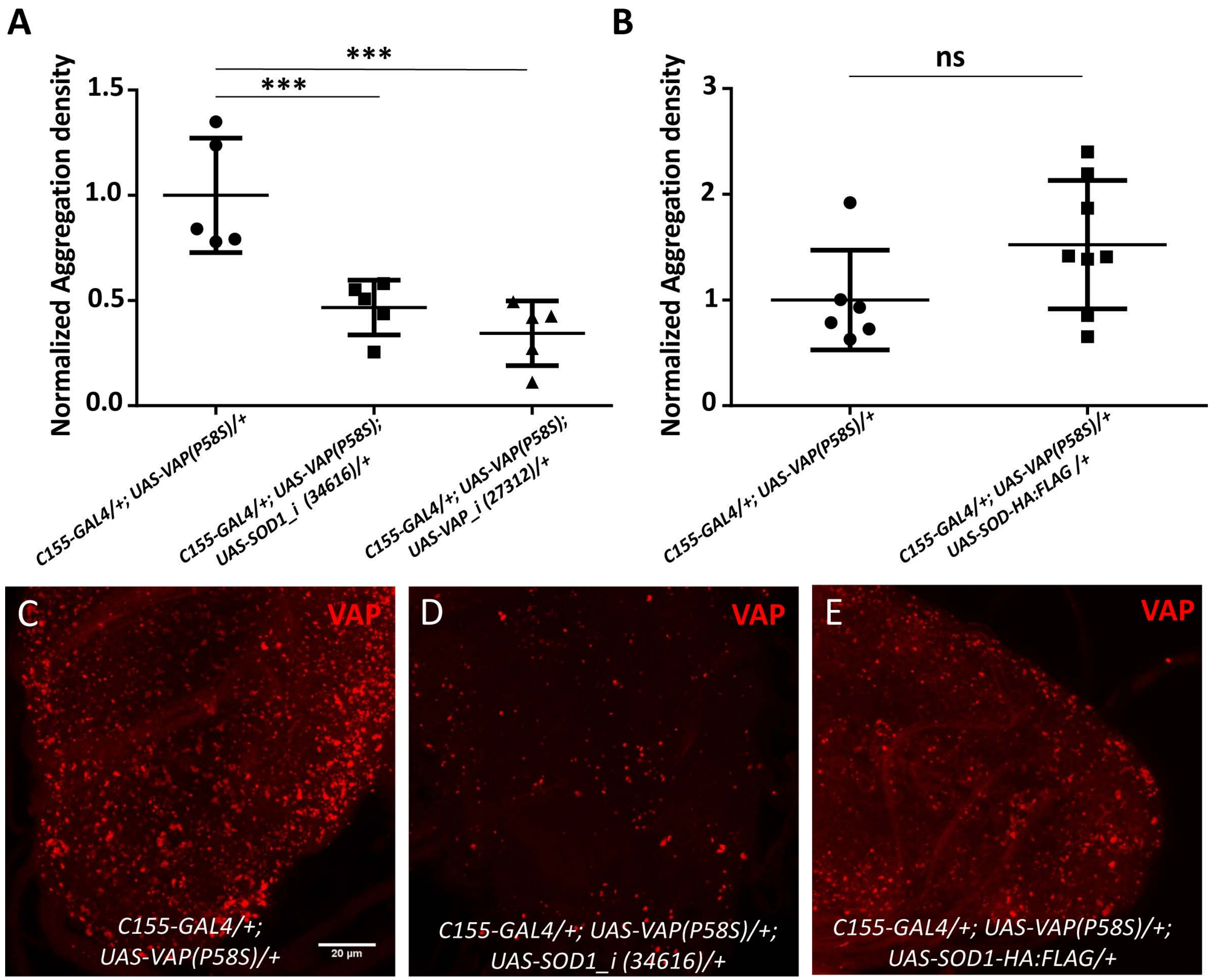

FIGURE S3: SOD1 modulates VAP(P58S) aggregation density in the third instar larval brain

A: *SOD1* knockdown decreases aggregation density. ANOVA (P-value ***, 0.0004) Fisher's LSD multiple comparison test (P-value, ***<0.001)

B: *SOD1:HA:Flag* overexpression does not affect aggregation density. Student's t test (P-value: 0.1066)

C, D, E: Representative images of the ventral nerve cord showing aggregation of VAP(P58S) **(C)**, with *SOD1* knockdown **(D)**, and with *SOD1-HA:Flag* overexpression **(E).** All images were taken at the same magnification. The '_*i*' appended to the gene name indicates a RNAi line with the number in brackets denoting a unique BDSC number. ANOVA (P-value: ****<0.0001) Fisher's LSD multiple comparison test (P-value, **<0.01, ***<0.001).

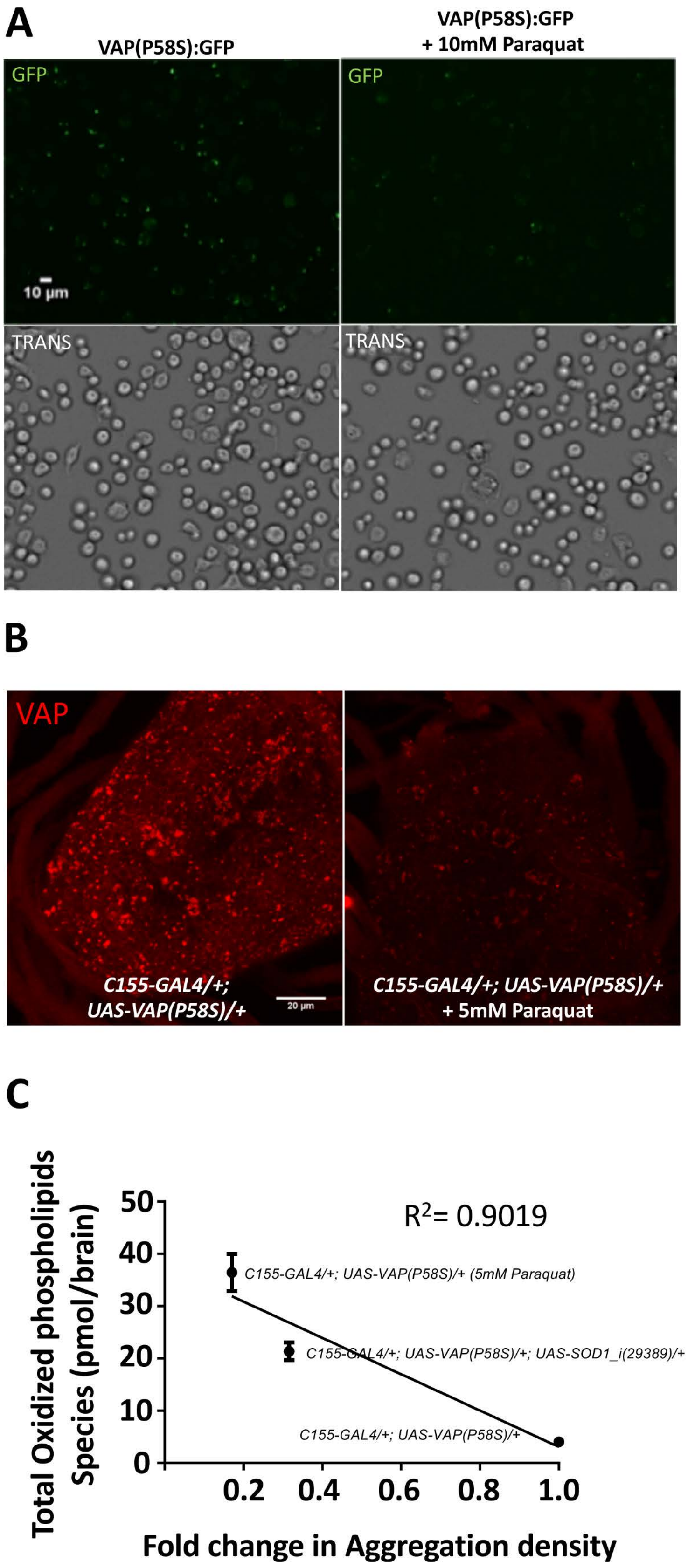

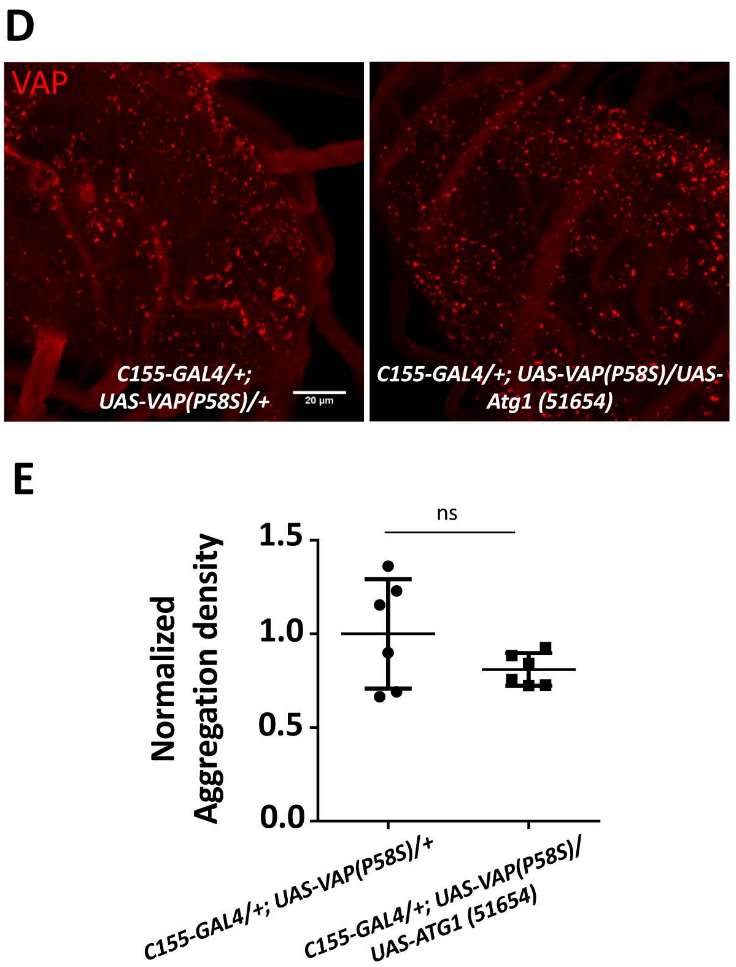

FIGURE S4: ROS induces clearance of VAP(P58S) aggregation, but not autophagy.

A: 10 mM Paraquat treatment for 4 hour, prior to inducing VAP(P58S):GFP in stable S2R+ cell line, reduces the fraction of cells showing aggregation observed 24 hours post-induction. Fraction of cell showing aggregation are plotted in Figure 4A.

B: Feeding 5 mM paraquat decreases aggregation density in the ventral nerve cord of third instar larval brains of *C155-GAL4/+; UAS-VAP(P58S)/+* flies. All images are taken at the same magnification. Aggregation density is plotted in Figure 4B.

C: Inverse correlation between total oxidized phospholipids and fold change in aggregation density.

D-E: Neuronal overexpression of Atg1 did not affect the aggregation density in the ventral nerve cord. Not Significant (ns), Students's t-test. All images were taken at the same magnification.

Disease Models & Mechanisms • Supplementary information

Table S1

A. List of 900 genes utilized for the screen. List is sorted alphabetically based on gene symbol.
B. 900 genes, utilized for the screen, classified and listed into 10 categories associated with ALS or VAP or proteostasis.
C. List of 150 modifiers of VAP(P58S) aggregation, based on average cell intensity, along with their human orthologs.
D. List of 85 modifiers of VAP(P58S) aggregation, based on total cell intensity, along with their human orthologs.
E. List of 57 common modifiers of VAP(P58S) aggregation, along with their human orthologs.

Click here to Download Table S1

Table S2

A. Details of the MRM transitions for the different phospholipids measured
B. LC-MS quantitation of the different phospholipids for different genotypes and paraquat treatment.
C. LC-MS quantitation of the different phospholipids for knockdown of *TOR*.

Click here to Download Table S2
